# The More the Better: Genetic Monitoring of *Paracentrotus lividus* (Lamarck, 1816) Experimental Restockings in Sardinia (Western Mediterranean Sea)

**DOI:** 10.3390/ani15040554

**Published:** 2025-02-14

**Authors:** Simone Di Crescenzo, Chiara Pani, Viviana Pasquini, Marco Maxia, Pierantonio Addis, Rita Cannas

**Affiliations:** 1 Department of Life and Environmental Sciences, University of Cagliari, 09126 Cagliari, Italy; simone.dicrescenzo@unica.it (S.D.C.); c.pani21@studenti.unica.it (C.P.); viviana.pasquini@unica.it (V.P.); 2 Agris, Agricultural Research Agency of Sardinia, Loc. Bonassai S.S. 291 Sassari-Fertilia—Km. 18,600, 07100 Sassari, Italy; mamaxia@agrisricerca.it

**Keywords:** cytochrome b, cytochrome oxidase subunit I, microsatellites, genetic diversity, Mediterranean Sea, common purple sea urchin, *Paracentrotus lividus*, Sardinia, restocking, genetic monitoring

## Abstract

*Paracentrotus lividus* is a widely distributed species in the Mediterranean Sea and North-East Atlantic Ocean, where it plays an important ecological and commercial role. The growing demand for its delicious gonads has rapidly led to the overexploitation of the natural populations around Sardinia (western Mediterranean). The present research aimed at understanding the population genetics of the species within the area, and at gathering data on the juveniles produced in an experimental ‘conservation hatchery’ facility. A multilocus approach was used, combining mitochondrial genes (COI and Cytb) and microsatellite markers. Overall, both the microsatellites and mitochondrial results indicate that the hatchery-produced juveniles were less genetically diverse and significantly divergent from the wild populations, most likely because of a bottleneck effect due to the insufficient number of parental breeders used. As concerns the wild populations, despite the high harvesting pressure, they still have a good quantity of genetic variation. A weak overall differentiation was found, suggesting extensive gene flow among the sites. However, the differentiation in the pairwise comparisons between the wild Sardinian samples was found to be significant with regard to the mitochondrial sequences, to be further investigated with additional studies. These data provide the scientific knowledge necessary to inform future management actions and to improve future aquaculture protocols.

## Simple Summary

The common purple sea urchin, *Paracentrotus lividus*, is a widely distributed species in the Mediterranean Sea and the North-East Atlantic Ocean. In recent years, it has been heavily exploited for human consumption, with a drastic reduction in its abundance and increasing interest in the experimental farming of it for commercial and restoration purposes. Using genetic tools, the present research aimed at characterizing the Sardinian wild populations and the genetic differences between wild and hatchery-reared individuals. A substantial reduction in genetic diversity and differentiation from the wild populations was found in the sea urchins from the hatchery, characterized by a very small genetic diversity as well as a high degree of relatedness. The results clearly indicate that the breeders used in the hatchery were insufficient to represent the wild populations; hence, larger numbers should be used in future rearing attempts. Overall, the wild Sardinian populations appear to be weakly differentiated, suggesting a large degree of gene flow between them.

## 1. Introduction

The common purple Mediterranean sea urchin, *Paracentrotus lividus,* is a widely distributed species in the shallow marine coastal rocky habitats of the Mediterranean Sea and North-East Atlantic Ocean, playing an important ecological role in controlling the macroalgal beds assemblages in benthic ecosystems [1,2,3,4,5]. Highly appreciated in the markets of many Mediterranean regions and in some other non-Mediterranean European areas [6], both males and females are harvested for their gonads, the so-called “roe” market [2]. In the last decades, a marked decline in its population in many European countries has been recorded due to human harvesting of this species, which has progressively intensified over the years ([7] and references therein). Consequently, the interest in maintaining *P. lividus* populations, both for ecological and commercial purposes, has rapidly grown [8].

In Italy, sea urchin fisheries are mainly located in the south and the main islands, including Sardinia (W. Mediterranean Sea), where several sea urchin fisheries were set up at the end of the 1980s and have steadily increased their production [2]. Given their significant social–economic impact, the autonomous government of Sardinia has imposed strict management rules to prevent their over-exploitation, such as limits on the number of firms authorized as fisheries, a defined harvesting season to respect their reproductive and growth periods, daily individual quotas, and a minimum size limit [9].

Despite these regulations, over the years legal and illegal fishery practices, for both commercial and recreational purposes, have caused wide and severe impacts on the natural populations ([2,6,7,8,10,11,12,13,14] and references therein). The growing demand from locals and tourists, as well as an increase in fishing efforts, has rapidly led to their overexploitation in some areas, with the populations largely depleted of commercial sizes ([2,6,7,8,10,11,12,13,14] and references therein). The various restrictions imposed by the Sardinia region to minimize the risk of over-harvesting have not allowed for the sea urchin populations to recover. The efficacy of the present management is dubious, being to date unsuccessful at conserving the stock and ensuring a sustainable fishery ([11,15] and references therein).

In general, the abundance of sea urchins has rapidly declined in the whole Mediterranean Sea and in the Atlantic due to the intense and targeted harvesting pressure; in addition, this species is negatively affected by pollution and global climate change [16,17,18,19].

Given the growing interest in the maintenance of *P. lividus* populations both for ecological and commercial aims [3,8,20], local and international research on echinoderm rearing (i.e., “echinoculture”) has gained popularity in Asian countries, and more recently in Europe ([5,21,22,23,24,25] and references therein). Such aquaculture facilities are mostly experimental, and they often struggle to be economically viable [20].

In Sardinia, aquaculture for commercial echinoculture is currently under development [3,21,26,27], with the main aim of providing the biomass to restore the depleted stock through repopulation programs, by releasing reared specimens into the environment [3]. In this context, the Department of Life and Environmental Sciences of the University of Cagliari (Italy) has established an experimental hatchery for echinoderms ([3,21,26,27] and references therein).

However, the question of whether the release into the wild of reared sea urchins could help to restore their populations has still to be explored properly. In particular, special attention has to be paid to the genetic impacts of reseeding hatchery-reared juveniles into wild populations, given that the releasing of large numbers of captive-bred animals into the wild can affect the genetic composition of local populations [22]. Therefore, a responsible approach to marine stock enhancement has been invoked [22,28,29,30], including, among others, the setting of clear genetic benchmarks and the use of appropriate brood stock and verified protocols to minimize the genetic impacts on wild populations [29,30].

At present, there is little published information concerning the genetic implications of reseeding sea urchins in Asian countries [22]. As concerns *P. lividus*, a few studies have investigated the impact of such practices on both the population structure and genetic diversity of wild communities, with contrasting results [31,32,33,34]. While [31,32] concluded that genetic diversity was not affected by introducing juveniles from a hatchery into wild populations, ref. [34] found hatchery-reared populations less diverse and significantly different from wild populations. In general, all the authors agreed on the necessity of the close genetic monitoring of the aquaculture strategies applied when restoring wild populations [22,28,29,30,32,34]. Stock enhancements should be accompanied by a comprehensive genetic monitoring program, including several steps as follows: (1) a baseline population genetics study of the recipient species in the area of interest to describe the level and distribution of genetic diversity; (2) the genetic identification of all broodstocks; (3) the genetic monitoring of the broods to compare their genetic diversity with that of the population into which they will be introduced; and (4) the monitoring of the admixed population and its wild-population component during the period of time over which the releases occurred and after they occurred [28]. Moreover, to minimize the risk of pathogen transmission from aquaculture plants to the wild population (e.g., Bald Disease [35]), the breeding facility must be equipped with adequate water treatment equipment to prevent disease spread [21]. Additionally, only healthy sea urchins, showing no signs of stress or disease, should be released after completing a quarantine period in the tanks. In addition, adequate studies on the control of pathogens (i.e., by analyzing dead individuals during rearing) are desirable before farmed sea urchins are released into the environment. In aquaculture, this kind of approach is most common for vertebrates and less so for invertebrates, excluding some case studies on shrimp (e.g., [36]).

Nowadays, genetic technologies are widely recognized as irreplaceable tools for fisheries’ stock identification, the genetic improvement of aquaculture species, and the characterization of ecosystem changes due to environmental and anthropogenic stresses ([37,38,39,40,41,42,43,44,45,46,47] and references therein). As concerns the focus of our study, for several echinoderms genetic data were obtained from the monitoring of wild populations and hatchery practices [48,49,50,51,52,53], and used to inform conservation management.

In this context, the present research aimed to present the first genetic monitoring of hatchery-reared juveniles in the experimental facility of the University of Cagliari and the nearby exploited *P. lividus* populations using a multilocus approach with a combination of mitochondrial genes (COI and Cytb) and microsatellite markers, extensively used in genetic studies of this species [1,4,31,32,34,54,55,56,57,58,59].

Therefore, we aimed (i) to obtain an understanding of the population history and current dynamics of *P. lividus* within the area and (ii) to gather data on the characteristics of the juveniles produced in the experimental ‘conservation hatchery’ facility, and on their possible use in future population restockings.

## 2. Materials and Methods

### 2.1. Sampling and DNA Extraction

**Figure 1:**
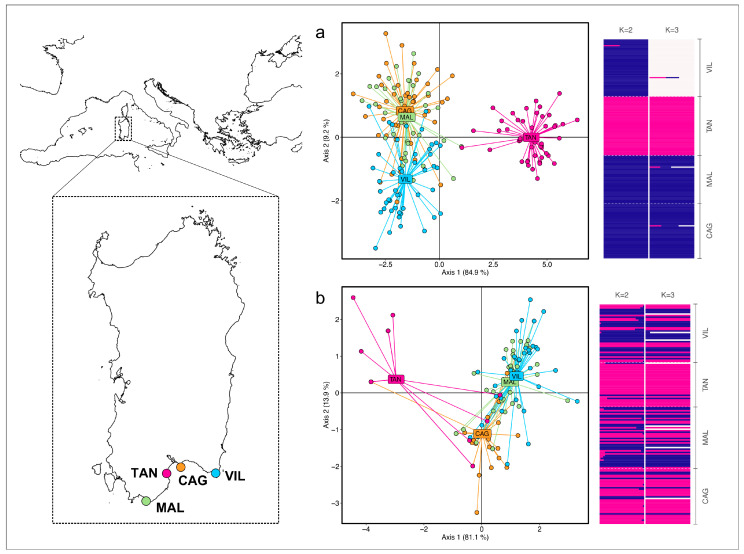
Map showing the position of *Paracentrotus lividus* samples. CAG = Cagliari; MAL = Malfatano; VIL = Villasimius; TAN = tanks in a hatchery facility near Cagliari. DAPC scatterplot for *P. lividus* samples and BAPS clustering barplots for (**a**) microsatellites and (**b**) mitochondrial data. Membership probabilities for each individual are shown for K = 2 and K = 3.

A total of 192 *P. lividus* specimens were collected in July 2023 in the western Mediterranean Sea (south Sardinian coast). Common purple sea urchins derived from three wild populations—Cagliari (CAG; n = 48), Capo Malfatano (MAL; n = 48), and Villasimius (VIL; n = 48)—and from a hatchery (n = 48; Figure 1, Table 1). Larval rearing and sea urchins’ growth of the species *P. lividus* were carried out in the facility as described by [21]. Laboratory procedures for larval rearing and tissue sampling and storage are described in the Supplementary Information document (henceforth SI).

DNA extractions were carried out with a DNeasy Blood & Tissue Kit (Qiagen, Hilden, Germany) following manufacturer’s protocol. The obtained gDNA from each sample was used to amplify nine microsatellite loci and two mitochondrial genes (COI and Cytb).

### 2.2. Microsatellite Data and Analysis

Individuals of *P. lividus* were genotyped at nine microsatellite loci: Pliv_Hist, Pliv_T, Pliv_15, Pliv_28, Pliv_F, Pliv_C, P_Liv_B, Pliv_32, and Pliv_L [60]. PCR conditions with primer pairs are described in Supplementary Information and Table S1, respectively. The microsatellites’ allele size was scored using GENEMARKER v.1.8 (SoftGenetics Inc.) after setting the proper panels for binning analysis. Micro-Checker v.2.2.3 was used to check the data for genotyping errors [61]. Null alleles frequency for each locus and sample was inspected with the program FreeNA [62], using the algorithm by [63]. Mean number of alleles (Na) and observed (Ho) and unbiased expected heterozygosity (UHe) were calculated using GenAlEx v.6.5 [64]. Allelic richness (Ar) and private allelic richness (Par) were estimated using the rarefaction method implemented in the software HPrare v.1.0 [65], based on the lowest sample size (33 individuals). All loci and samples were tested for linkage disequilibrium (LD) and deviations from Hardy–Weinberg expectations (henceforth HWE; null hypothesis H1 = heterozygote deficiency) using exact tests in GenAlEx. The inbreeding coefficient *F*_IS_ was computed over all loci for each sample and for single locus over all samples with GenoDive v. 3.04 [66].

The software ML-relate [67] was used to infer genealogical relationships amongst individuals. It is a maximum likelihood-based software that estimates relatedness coefficients (R) for each pair of individuals and provides the relationships that have the highest likelihood for each pair of individuals (PO = parental-offspring, HS = half-sibling, FS = full-sibling, and U = unrelated). 

Genetic differentiation among populations was investigated based on pairwise fixation indices calculated with 10,000 permutations using Arlequin v 3.5.2.2 [68]. The null allele impact on the fixation index *F*_ST_ was evaluated by calculating *F*_ST_ with and without excluding null alleles using the ‘excluding null allele’ (ENA) correction implemented in FreeNA.

The occurrence of population structuring within the studied area was investigated using multiple approaches (see Supplementary Information for details): (1) Bayesian clustering with BAPS v.6 (Bayesian Analysis of Population Structure) [69,70,71]; (2) Discriminant Analysis of Principal Components (DAPC [72]) using the R package Adegenet v.2.1.10 [73,74] implemented in R v.4.3.1 [75]; and (3) analysis of molecular variance (AMOVA) in Arlequin.

Microsatellite data were checked for the occurrence of recent demographic changes, namely genetic bottlenecks, using the software BOTTLENECK v.1.2.02 [76] (see Supplementary Information for details).

NEESTIMATOR v.2.1 [77] was used to calculate the effective population size (Ne) and the number of effective breeders (Nebs) for each sampling site. The LD method and the molecular coancestry method of [78] were used to evaluate Ne and Nebs, respectively.

To reconstruct source–sink population dynamics and the evolutionary processes leading to the present genetic diversity distribution, relative migration rates among wild populations were estimated based on allele frequency data according to the method of [79] using the divMigrate function implemented in the R package diveRsity [80].

### 2.3. Microsatellites Data: Comparison with Previous Studies

To confront the genetic features of Sardinian populations with other areas, previous studies based on the same markers were scrutinized [31,32,34,59]. To avoid erroneous conclusions when making comparisons with previous studies based on different sets of microsatellites [31,32,34,59], all the overall statistics in our dataset (Na, Ar, Ho, He, *F*_ST_, and *F*_IS_) were recalculated to match the markers used in the original publications. To assess the differences in the main indices, the Kruskal–Wallis nonparametric statistical test [81] implemented in XLSTAT [82] was used. The *p*-value was computed with Monte Carlo method and 10,000 simulations.

### 2.4. Mitochondrial Data and Analysis

The primers for the amplifications of Cytb and COI were obtained from [4,83], respectively (see Supplementary Information for details).

The new COI and Cytb sequences were imported into MEGA 12 [84], carefully edited, and aligned with the CLUSTAL W algorithm [85].

DNASP v.6 [86] was used to (a) collapse the sequences into haplotypes and (b) estimate the principal indices for mtDNA (the number of haplotypes [H], haplotype diversity [hd], nucleotide diversity [π], and relative standard deviations).

The reconstruction of haplotype networks and the relationships among haplotypes were realized with the TCS method [87] implemented in the software Hapsolutely v.0.2.2 [88].

The partition homogeneity test [89] implemented in PAUP* v.4.10 [90] was used to test if the COI and Cytb sequences could be concatenated.

The software Arlequin v3.5.2.2 was used to estimate pairwise fixation indices Φ_ST_ values calculated from sequence divergences using the best models identified with MEGA (see Supplementary Information for details). The significance of fixation indices values was computed by a nonparametric permutation procedure with 10,000 iterations.

Similarly to the microsatellite data, the occurrence of population structuring was investigated using BAPS, DAPC, and AMOVA (see Supplementary Information for details). The output of the admixture clustering analysis was used to graphically represent the results using the POPHELPER online tool [91].

To reconstruct historical demography, a mismatch distribution analysis [92] was computed on DNASP v.6 under the “Constant Population Size” and “Population Growth-Decline” models. Additionally, the sums of squared deviations (SSDs) and Harpending’s raggedness r [93] observed versus that which was expected based on two expansion models (the sudden demographic expansion and spatial expansion models) were computed in Arlequin v 3.5.2.2. Additionally, the historical demography of the *P. lividus* populations was examined using three statistics: Tajima’s D [94], Ramos-Onsins’ R2 index [95], and Fu’s FS [96]. All these statistics were computed in DNASP v.6, and their significance was tested with 1000 coalescent simulations under the Standard Neutral Mode. An additional method for determining historical demography was used, the Coalescent Bayesian Skyline [97,98]. The Bayesian Skyline plot analysis (BPS) was carried out using Beast v.1.10.4 [99]; further details are provided in SI.

### 2.5. Mitochondrial Data: Comparison with Public Sequences

In order to investigate the occurrence of genetic differentiation among Mediterranean, Atlantic, and other areas of its wide distribution range, the sequences for *P. lividus* obtained in this study were compared to homologous sequences available from GenBank (https://www.ncbi.nlm.nih.gov/genbank, accessed on 13 September 2024) [100] and BOLD [101] (http://www.boldsystems.org, accessed on 13 September 2024) databases. A total of 144 and 261 sequences were retrieved for COI and Cytb, respectively. A genetic admixture analysis was used to evaluate the presence of population genetic structure using a Bayesian model-based clustering algorithm implemented in BAPS v6. The BAPS haplogroups were visualized as pie charts on a map using GENGIS 2.5.3 [102]. The European coastline shapefile used to produce the maps was downloaded from the EEA website (https://www.eea.europa.eu/data-and-maps/data/eea-coastline-for-analysis-1/gis-data/europe-coastline-shapefile, accessed on 13 September 2024). The occurrence of population structuring was further tested using AMOVA in Arlequin.

## 3. Results

### 3.1. Microsatellites Results

#### 3.1.1. Microsatellite Variability

Common purple sea urchins from three wild locations and experimental hatchery facility were initially genotyped at nine loci. However, the locus Pliv_F was problematic: it failed to amplify in a high number of individuals in the tank-reared sea urchins and, hence, it was removed. The final dataset for *P. lividus* consisted of 179 individuals genotyped at eight loci. After an FDR correction, six loci were found to be in LD, but mainly in the TAN sample; therefore, all the loci were maintained for the final analysis. The statistics for the microsatellite data for both the samples and loci are described in Table 1, Tables S2 and S3.

The microsatellite loci showed a higher polymorphism in the natural populations than in the tank-reared individuals (Table 1 and Table S3). The mean number of alleles (Na) per sample was quite similar for all the wild sites (range 19.25–21), while lower values were recorded for the tank (8.38). Similarly, the allelic richness (Ar) reached the highest value at MAL (19.92) and the lowest in the TAN (7.99). Significant deviations from Hardy–Weinberg equilibrium (HWE), measured as a deficiency in heterozygotes, occurred at all eight loci and in all the samples. In addition, most of the population-by-locus combination tests showed deviations from HWE. The deficit was usually generated by the presence of null alleles, population substructuring, or inbreeding. In our study, the inbreeding coefficient *F_IS_* ranged from 0.46 to 0.55, being significantly higher than zero at all sites. The proportion of null alleles (Fnu) ranged from 0.177 (Pliv_C) to 0.471 (Pliv_L), with an average of 0.25 (Table S2). According to [62], this estimate can be classified as moderate (0.05 < r < 0.20) to high (r > 0.2). After the removal of the two loci with the highest null alleles frequencies (r > 0.25: Pliv_38 and Pliv_L), the results of all the analyses did not change. Therefore, all the loci were retained for the subsequent analyses, consistent with previous studies where the same loci with null allele frequencies were employed for studying purple sea urchins [31,34].

#### 3.1.2. Genetic Differentiation and Population Structure

The genetic relationships among the samples investigated are presented as a matrix of the pairwise *F_ST_* and *D_EST_* (Table 2). The pairwise values of both indices were low or high, ranging from 0.002 to 0.100 (*F_ST_*) and from 0.061 to 0.582 (*D_EST_*). The only statistically significant comparisons, even after correction for multiple testing, were those involving the TAN. Low and not significant values were recorded for the comparisons involving the wild samples. The same result was obtained for the estimation of the pairwise *F*_ST_ with an ENA correction for the null alleles (Table S4).

The Bayesian clustering analysis in BAPS indicated the best scenario at K = 2 (highest marginal likelihood value; log(mL) = −8057.4152), grouping all the locations except the TAN, immediately followed by K = 3 (log(mL) = −8259.4445), corresponding to the three clusters of the TAN, VIL, and CAG + MAL (Figure 1). The DAPC yielded overall congruent results, suggesting the occurrence of three clusters (Figure 1).

The AMOVA analysis showed a global *F_ST_* = 0.054 (*p* = 0.000), rejecting the hypothesis of genetic homogeneity. However, less than 5.40% of the total variance was among the samples and 94.60% was within the samples. The AMOVA, conducted based on the clusters previously identified by BAPS, did not confirm a significant level of structuring (two groups: *F*_CT_ = 0.088, *p* = NS; three groups: *F*_CT_ = 0.0605, *p* = NS; Table S5). Lower values for the overall *F_ST_* were detected with an ENA correction for the null alleles (0.037).

The directional relative migration networks based on the *G_ST_* estimates of *P. lividus* populations reconstructed with *divMigrate* show that gene flow exists among the studied localities (Figure S1). No significant directional migration was observed.

#### 3.1.3. Relatedness of Population Size and Demography

The ‘relatedness’ analysis (maximum likelihood method in ML-RELATE) estimated the percentage of first- and second-degree relatives (FS + HS) to be low in the wild population samples, from 2.93% at CAG to 3.70% at VIL, but quite higher in the TAN (16.40%) (Table 3).

The lowest estimates for the number of effective breeders and contemporaneous Ne were recorded in the TAN, followed by VIL (Table 4). Apart from the TAN and VIL, infinite estimates were recorded at the other two wild locations (CAG and MAL) (Table 4). The bottleneck failed to show statistical evidence of a recent reduction in population size for the *P. lividus* samples, except at CAG (Table 1).

#### 3.1.4. Comparisons with Other Areas

Significant differences in the main indices were found using the Kruskal–Wallis nonparametric statistical tests; in most of the comparisons, the *F*_IS_ and Ho values were higher and lower, respectively, in the wild Sardinian samples than in the other areas (Table S6). In general, no significant differences were found in the Na, Ar, and He values.

### 3.2. Results: Mitochondrial DNA

#### 3.2.1. Mitochondrial Genes’ Variability

A 623 bp fragment of COI was obtained for 189 individuals, revealing a total of 60 haplotypes, while 146 Cytb sequences of 1018 bp resulted in 69 haplotypes (Tables S7 and S8). The haplotypes for the individual markers were deposited into GenBank (accession numbers: COI—PQ796658–PQ796717; Cytb—PQ801498–PQ801566). The partition homogeneity test [89] indicated that the two datasets (COI and Cytb) did not significantly differ in their phylogenetic signals (*p* = NS); therefore, a concatenated COI-Cytb alignment of 143 sequences was created (length: 1641 base pairs). If not specified differently, the concatenated sequences (Table S9) were used for the measure of the diversity indices as well as all the demographic inferences, because of the presumed statistical advantages of longer datasets [103]. Table 5 shows the principal indices for the combined markers, while the values for the single genes are in Table S10. The combined COI + Cytb sequences show 107 haplotypes and, in general, the samples are characterized by a high haplotype diversity (hd_COI+Cytb_ = 0.984; hd_COI_ = 0.939; and hd_Cytb_ = 0.925) and low nucleotide diversity (π_COI+Cytb_ = 0.009; π_COI_ = 0.007; and π_Cytb_ = 0.010). Considering the combined genes, the haplotype diversity (hd) among the samples varies from 0.709 (TAN) to 1 (VIL), while the nucleotide diversity ranges from 0.003 (TAN) to 0.010 (VIL).

**Figure 2:**
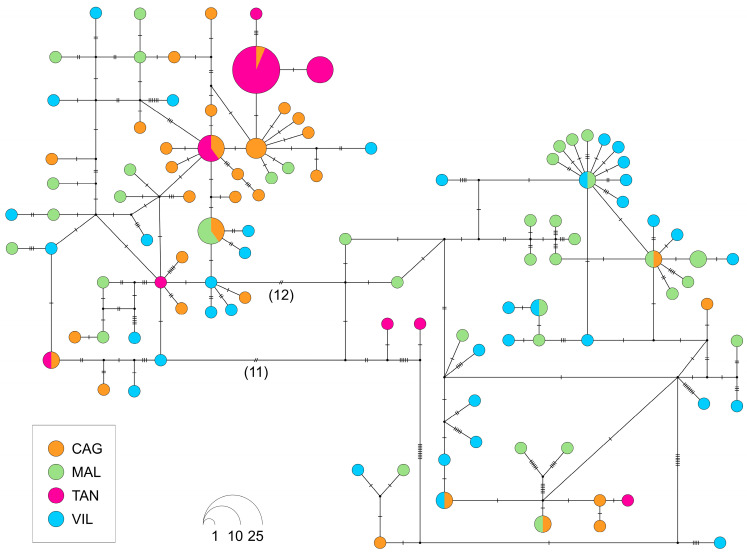
Haplotype network realized with the TCS method using the COI + Cytb sequences. The size of the pie charts is proportional to the corresponding haplotype frequency, while the color indicates the geographical origin. The dots indicate inferred unsampled haplotypes, while the lines indicate single substitutions. The abbreviations are in accordance with those in Figure 1. The number near the lines indicates the number of mutations.

The haplotype network for the concatenated sequences (Figure 2) illustrates the distribution of haplotypes among the four samples and their relationships. It is quite complex, with a predominance of singletons or haplotypes at low frequencies. The segregation into two main groups is evident, but it does not correspond to any strict geographic segregation.

#### 3.2.2. Population Differentiation and Structure

All the overall fixation indices (Φ_ST_) indicate a low but significant differentiation among the samples (Table S5: COI + Cytb—Φ_ST_ = 0.218, *p* = 0; Table S11: COI: Φ_ST_ = 0.250, *p* = 0; and Cytb—Φ_ST_ = 0.211, *p* = 0).

The pairwise population comparisons point out that the differentiation involves most samples. As concerns the concatenated mtDNA sequences, the Φ_ST_ values (range 0–0.399) are significant for all comparisons except MAL/VIL, even after an FDR correction (Table 2). Similar results are obtained for the COI sequences’ Φ_ST_ values (range 0–0.46), while for the Cytb, along with MAL/VIL and CAG/TAN, they were not significantly different (Table S12).

The BAPS Bayesian clustering analysis using the concatenated dataset indicates the best scenario at K = 2 (highest marginal likelihood value; log(mL) = −2712.583), grouping all the locations except the TAN (Figure 1). The DAPC suggests a distinction between the TAN and CAG from MAL + VIL (Figure 1).

The AMOVA based on the clusters previously identified by the BAPS and DAPC does not confirm a significant level of structuring (Table S5: COI + Cytb—two groups: Φ_CT_ = 0.180, *p* = NS; three groups: Φ_CT_ = 0.275, *p* = NS) (see Table S11 for the single genes).

For the concatenated dataset, the historical demographic analyses provide controversial signals (Table 6, Figure S2). The mismatch distributions are clearly bimodal in the three wild populations, as well as when they are combined together in a single dataset (“wild”, Figure S2), with a shape like the one expected for stationary populations or in cases of contact between two or more different populations. All the different tests coherently indicate expansion/growth in the ‘wild’ sample, but not in the single samples (Table 6). The significant Fu’s FS [96] neutrality tests reject the hypothesis of neutrality, while the non-significant values for Harpending’s raggedness index [93], as well as the sums of the squared differences, does not allow for the rejection of the expansion models. However, the D values are not significant for all sites, similarly to the R2 values at MAL and VIL, and do not allow for the rejection of the hypothesis of neutrality (stationarity) (Table 6).

**Figure 3:**
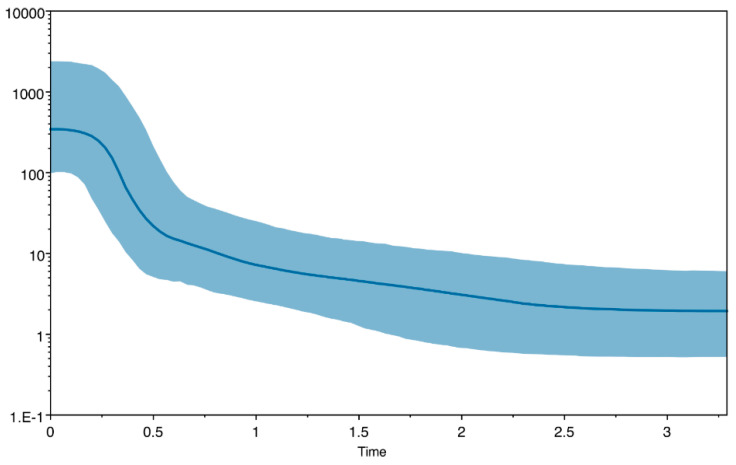
Bayesian Skyline plot using the COI + Cytb sequences. The x-axis represents time in MYA (millions of years ago), while the y-axis shows the effective population size. The thick blue line represents the median, while the blue band represents the 95% highest posterior density (HPD) intervals.

The Coalescent Bayesian Skyline analysis suggests that the population size changes are characterized the recent population history of *P. lividus* (Figure 3). The BPS shows a rapid population size growth dated to back between 250,000 and 500,000 YBP.

#### 3.2.3. Comparison with Other Areas

The Sardinian samples were compared to all the available sequences for a given gene to evaluate the relationships within the species *P. lividus*.

For the COI gene, 189 sequences from Sardinia were compared with 144 *P. lividus* homologous public sequences (Table S7) from different geographical areas.

The final alignment counted 333 sequences (601 bp) and 114 haplotypes. In the haplotype network (Figure S3), a complex star-like shape is visible, with the most common haplotypes shared among several locations, included both in the Atlantic and Mediterranean Sea. The BAPS Bayesian assignment analysis identified four distinct haplogroups (Figure S4) occurring at different frequencies in all the samples except for Haplogroup D (pink in Figure S4), which is the most common in the Mediterranean (40.84% of individuals), but totally absent in the Atlantic samples.

The pairwise population comparisons point out that the differentiation involves the TAN, CAG, and the Atlantic locations, as well as the easternmost site (EGY, Figure S5). The AMOVA results indicate the occurrence of significant genetic differentiation between the Atlantic and Mediterranean sites (Table S11).

As concerns the Cytb gene, 146 sequences from Sardinia were compared with 261 *P. lividus* homologous public sequences (Table S8) from different geographical areas. The final alignment counted 407 sequences (1018 bp) and 213 haplotypes. The haplotype network (Figure S6) is quite complex, with a few shared haplotypes and many singletons. The BAPS Bayesian assignment analysis identified three distinct haplogroups (Figure S7) occurring at different frequencies in all the samples. Haplogroup C (blue in Figure S7) is the most common in the Atlantic samples, Haplogroup A (yellow in Figure S7) is the most abundant in the Adriatic samples, and Haplogroup B (pink in Figure S7) is the most widespread in the Mediterranean (37.84% of individuals).

The pairwise population comparisons point out that the differentiation involves the TAN and CAG vs. the Atlantic, Adriatic, and easternmost sites (Figure S8). The AMOVA results indicate the occurrence of significant genetic differentiation between the Atlantic and Mediterranean sites, and within the Mediterranean between the CW Mediterranean sites and the Adriatic (Table S11).

## 4. Discussion

The protection of genetic diversity and adaptive potential in wild populations must be primary concerns of any supplementation program, because of the potential for harming not only the target species, but also the community of individuals interacting with the target species ([30] and references therein).

Several authors agree on the fact that genetic risks from stock enhancements can be substantial because of (1) inattention to brood stock sizes that can lead to the loss of genetic diversity, and (2) hybridization between hatchery-reared and wild individuals that can lower the fitness or lead to the extinction of a natural population [30].

In this context, this study describes the first genetic monitoring of *P. lividus* populations on the Sardinian coasts, both natural populations and hatchery-reared individuals, using multiple markers.

### 4.1. Wild vs. Hatchery-Reared Sea Urchins

Overall, both the microsatellites and mitochondrial results indicate that the hatchery-produced juveniles are less genetically diverse and significantly divergent from the wild populations. Similar results were obtained in the Atlantic by [34] when they compared the levels and patterns of genetic variation between two hatcheries and four nearby wild populations from Spain and Ireland. The farmed sample has fewer haplotypes, low numbers of alleles, a very small effective population size, and a higher degree of relatedness between individuals. In particular, the estimated effective population size (Ne) is very reduced, varying from 3 individuals to 12 individuals (Pcrit = 0.01; Table 4), suggesting that they originated from only a few spawning individuals. The Ne value is largely lower than that measured in previous studies of urchins hatcheries [32,34], and well below the minimum value recommended by most authors to avoid genetic problems in the short term (Ne = 50; [34]). Such a small effective population size implies that random genetic drift has a greater influence than natural selection, and therefore favorable alleles can decline in frequency and deleterious alleles may increase by chance [34].

Our results reconfirm the findings of other genetic studies on urchin hatcheries in Europe and Asia: the genetic differentiation occurs in the first generation of the hatchery-produced juveniles ([34] and references therein).

The genetic divergence between the hatchery and wild populations indicates that the hatchery sample is not representative of the wild gene pool, most likely because of a bottleneck effect due to the use of an insufficient number of breeders and/or the use of brood stock from an overexploited area (Addis and Pasquini, personal communication). Actually, a large proportion of full-sibs and half-sibs were found in the Sardinian hatchery sample with respect to the wild populations. Similarly, ref. [32] measured high relatedness values in the hatchery-produced offspring in the Gulf of Biscay. Bottlenecks are known to occur also in the wild, given that broadcast spawners, such as the sea urchin *P. lividus,* are characterized by a high fecundity with the release of over a million eggs per female, but only a few survive up to fertilization and settlement [104,105]. Similarly, among the several steps for *P. lividus* breeding, the main bottleneck is the mortality observed during the larvae settlement and metamorphosis, which is characterized by 5 to 10% of post-larvae survival under rearing conditions [106].

The Sardinian hatchery output (produced with the current protocol) was not optimal and not able to maintain the natural levels of genetic diversity, resulting in an increase in relatedness and significant changes in allele frequencies in the cultured juveniles. Therefore, the releasing of a large number of hatchery-reared juveniles of *P. lividus* with such a low diversity would not be advisable, since it could pose a risk to the nearby recipient wild populations, possibly causing a reduction in genetic diversity and fitness [34]. However, opposite conclusions were reached by [32] when comparing wild samples with those subjected to restoration experiments with hatchery-reared urchins; the latter did not show significant negative genetic effects after the restocking actions.

### 4.2. Characteristics of Wild Sardinian Samples

Using SSRs, a weak overall differentiation was found, but the pairwise comparisons between wild Sardinian samples were always non-significant, suggesting an absence of barriers to gene flow in the investigated area. These results agree with most of the previous studies suggesting a panmictic scenario in *P. lividus* at small spatial scales, both in the Atlantic and in the Mediterranean, using microsatellites markers [1,34].

This pattern can be easily explained by the high potential for dispersal in this species: although its benthic adult life stage is sedentary, it can travel long distances during its planktonic larval period of 20–40 days, potentially ensuring gene flow between distant populations [34,107]. High and consistent potential connectivity between Sardinia, Corsica, and the coastlines of the Ligurian and Tyrrhenian Seas was measured for *P. lividus* larvae by [18], resulting in a lack of genetic structuring within the edible sea urchin population in this area.

As in previous studies on this species, we observed a deficit of heterozygotes relative to what would be expected for populations at Hardy–Weinberg equilibrium (Table 1), coincident with the results of [1,32,34]. It is possible that null alleles are the main cause of these deviations, being quite common in echinoderms and sea urchins ([1] and references therein).

However, positive assortative mating, inbreeding, selection, or genotyping errors could also explain the observed Hardy–Weinberg disequilibrium [58,107]. Other ecological processes, such as a recent admixture or temporal Wahlund effect, have also been commonly associated with heterozygote deficiency in sea urchins ([34] and references therein).

Compared to previous studies in other areas, the Sardinian populations were characterized by significantly lower values of Ho and higher values of *F*_IS_ (Table S6). In particular, the VIL sample showed the lowest observed heterozygosity values and the highest inbreeding, suggesting that it could be sustained through the local recruitment of related individuals [32]. The bottleneck analysis revealed a significant deficit in the expected heterozygosity of one sampling site (CAG), suggesting that this population could have undergone a recent demographic erosion. Such condition could probably be related to the heavy fishing pressure historically occurring in this area (Addis and Pasquini, personal communication).

The mitochondrial data showed a high degree of polymorphism in the Sardinian wild samples, with high levels of haplotype diversity and low levels of nucleotide diversity, and many haplotypes occurring at very low frequencies in both genes (COI and Cytb). This combination has been seen in previous studies [4,54]. It has been suggested that the occurrence of a large number of low-frequency haplotypes can result from the enormous population sizes of marine organisms, causing the retention of numerous haplotypes during population growth or expansion [54]. It has been hypothesized that *P. lividus* expanded in the Mediterranean after the last glacial maximum from donor populations with enormous population sizes. This should have favored both the existence of many rare haplotypes and an excess of rare mutations [55]. In the present study, almost all the neutrality statistics calculated from the data showed negative values, indicating past expansion or bottleneck recovery, confirmed also by the BPS plot. The bimodality observed in the mismatch distributions can be seen as a result of the presence of the different haplogroups detected, rather than of demographic stability [4].

Despite the high harvesting pressure, the populations of *P. lividus* in Sardinia still have a good quantity of genetic variation. COI was more variable and discriminant than Cytb; both markers have proven in previous studies to be highly polymorphic in *P. lividus* as well as in different echinoid species ([4,32,54,56] and references therein).

Including in our analyses previously published genetic data by [4,54] for the western and eastern Mediterranean, Atlantic, and Adriatic, the results suggest the occurrence of three–four main haplogroups at different frequencies. Our results are in line with previous studies based on mitochondrial markers that identified a few main genetic discontinuities between the Atlantic and the Mediterranean and between the Adriatic Sea and the rest of the Mediterranean Sea [4,54,55,56,57,107].

The microsatellites and mitochondrial data provided contrasting evidence as concerns the differentiation of the Sardinian samples. Both mitochondrial genes, singularly and in combination, identified significant differentiation among the Sardinian populations. In particular, CAG was significantly different from other close locations in southern Sardinia (i.e., MAL and VIL, about 40 km distant from CAG), as well as from distant locations located in the northwestern and northeastern coasts (ALG and PIT, Figure S7 and Table S8 [4]). This was rarely observed in previous studies based on mitochondrial markers at this spatial scale. However, the main limitations of past studies could have been the use of a single locus and sometimes very small sample sizes (e.g., in general, n = 10 per location in [4]), not allowing for the identification of significant genetic substructuring [56].

Within-basin and within-region differentiation was also measured within areas in which no stable or identified oceanographic barriers had ever been reported by [56], with the easternmost population of Lebanon strongly differentiated from other Mediterranean populations. Recently, ref. [32] described the Gulf of Biscay as different from the Atlantic *P. lividus* populations. Similarly, using SNPs, ref. [108,109] detected weak but significant differentiation between the Adriatic and western Mediterranean, as well as subtle patterns of differentiation within the Atlantic and Mediterranean Sea.

A peculiar result of this research is the contrasting pattern that has emerged from the analyses based on the microsatellites and mitochondrial data, with only the latter revealing significant genetic differentiation among the Sardinian wild populations. Similarly, ref. [109] found discrepancies when using nuclear vs. mitochondrial data for analyzing the Adriatic–Ionian populations, with SNPs indicating homogeneity and Cytb showing a significant differentiation among the populations in the area [4,56].

In general, it is unlikely that mtDNA markers could detect differences that do not emerge from nuclear markers. However, the uniparental inheritance makes mtDNA more strongly affected by genetic drift ([109] and references therein). The contrasting pattern involving CAG could be caused by selection or, perhaps, by the strongly skewed reproductive success of females. In fact, the successful reproduction of some females and the subsequent recruitment of their offspring would quickly increase the maternal haplotype frequency, leading to inflated differentiation [109]. It cannot be ruled out that some environmental or hydrological factors, relatively stable over the years, could probably be involved in the genetic differentiation found [56]. Although the prolonged larval stage of *P. lividus* is expected to facilitate high levels of gene flow as marine currents could drive the circulation of larvae (i.e., plutei) between distant localities, it is also possible that complex oceanographic features may disrupt gene flow and promote population subdivision [110].

Additional data over multiple years are required to better understand the peculiarity of the CAG sample, to monitor this site that is characterized by historically high fishing pressure.

## 5. Conclusions

Our research aimed at both the genetic characterization of the hatchery-produced juveniles and the elucidation of the population genetic structure of *P. lividus* in South Sardinia. The hatchery-reared urchins were characterized by a very small genetic diversity and high degree of relatedness compared to the wild Sardinian populations. Our preliminary findings provide scientific background knowledge to inform future management actions and to improve aquaculture protocols.

Here, we provided preliminary data on genetic monitoring, as it was included as a side program of the original restocking program, and realized only in a later step using residual marginal funds. Therefore, the characterization of the parental brooders was not possible nor the characterization of the wild population from which they were collected, since they were not available anymore at the time of the present analyses.

In the future, it is highly recommended that genetics be taken into account in the restocking program from the beginning to reduce the genetic risks associated with restocking practices. The hatchery procedures should be priorly genetically monitored to ensure that the hatchery-reared individuals represent the wild sea urchin gene pool and for the early detection of possible effects on the wild gene pools due to restoration efforts [32].

In particular, the present results clearly indicate that, in future protocols, a larger number of autochthonous breeders should be used to preserve a high genetic diversity in the restored populations, like the one observed in wild populations.

Furthermore, as suggested in previous studies [32], understanding the historical and contemporary population genetic diversity patterns of the declining *P. lividus* population are essential to better preserve the species gene pools under the threat of climate change, potential diseases, and overexploitation. Therefore, an extensive genetic analysis of all the wild populations along all the Sardinian coasts is urgent and should not be postponed.

## Figures and Tables

**Table 1:** Microsatellites descriptive statistics for *Paracentrotus lividus* samples: *w* = wild; *h* = hatchery-reared; n = number of individuals genotyped; Na = mean number of alleles; Ar = mean allelic richness; PAr = mean private allelic richness; Fnu = null alleles frequency; Ho = observed heterozygosity; uHe = unbiased expected heterozygosity; *F*_IS_ = inbreeding fixation index; HWE = probability for the multilocus Hardy–Weinberg test when H1 = heterozygote deficit; *** = *p* < 0.001. In bold are shown the significant values. TPM = probability of Wilcoxon’s signed-rank test for heterozygosity excess using two-phase model (TPM). Wild = CAG + MAL + VIL.

Sample	Origin	Code	n	Na	Ar	PAr	Fnu	Ho	uHe	*F*_IS_	HWE	TPM
Cagliari	w	CAG	45	21.000	19.890	2.330	0.247	0.502	0.933	**0.465**	***	**0.014**
Malfatano	w	MAL	39	20.375	19.920	2.140	0.246	0.507	0.925	**0.455**	***	0.422
Villasimius	w	VIL	47	19.250	18.180	1.570	0.257	0.468	0.927	**0.498**	***	0.809
Tank	f	TAN	48	8.375	7.990	0.060	0.252	0.345	0.763	**0.551**	***	---
Wild mean			114	26.875	19.330	2.013	0.250	0.492	0.929	**0.463**	***	0.230
SD			±0.895	±0.996	±0.396	±0.006	±0.036	±0.005	±0.039	**±0.895**		
All mean			43.469	17.250	16.495	1.525	0.250	0.455	0.887	**0.480**	***	---
SD			±0.758	±1.146	±5.728	±1.029	±0.005	±0.034	±0.887	**±0.037**		

**Table 2:** Pairwise fixation indices calculated for microsatellite loci (*F*_ST_/*D*_EST_) and the concatenated sequences (Φ_ST_). Negative values have been replaced with zero. The bold values are significant after FDR correction. *** = *p* < 0.001; ns = not significant.

***F*_ST_/*D*_EST_**	**CAG**	**MAL**	**TAN**	**VIL**
CAG		0.061	**0.582**	0.097
MAL	0.002		**0.531**	0.117
TAN	**0.097**	**0.093**		**0.578**
VIL	0.006	0.008	**0.100**	
**Φ_ST_/*p*-Values**	**CAG**	**MAL**	**TAN**	**VIL**
CAG		***	***	***
MAL	**0.399**		***	ns
TAN	**0.131**	**0.206**		***
VIL	**0.396**	0.000	**0.205**	

**Table 3:** Relatedness by maximum likelihood (R) using ML-relate software across *Paracentrotus lividus* samples. FS = full-sibling; HS = half-sib; PO = parent/offspring; and U = unrelated; second-grade relationship percentage = (FS + HS) %. R = the relationship with the highest likelihood estimated.

Sample	% of Individuals in Each Relationship in R				%
	FS	HS	PO	U	(FS + HS)
CAG	0.51%	2.42%	0%	97.07%	2.93%
MAL	0.27%	2.70%	0%	97.03%	2.97%
VIL	0.37%	3.33%	0%	96.30%	3.70%
TAN	8.42%	7.98%	0%	83.60%	16.40%

**Table 4:** NEESTIMATOR results. Effective population size (Ne) and the number of effective breeders (Nebs) for each sample. Ne values are given for 0.05, 0.02, and 0.01 thresholds for the lowest allele frequency used (Pcrit). The values in parentheses are the 95% confidence intervals based on jackknifing on loci. Sample codes are the same as in Table 1.

	Ne			Nebs
Sample/Pcrit	0.05	0.02	0.01	
CAG	inf (8.9–inf)	inf (12.0–inf)	inf (12.0–inf)	inf (inf–inf)
MAL	inf (5.6–inf)	inf (9.0–inf)	inf (9.0–inf)	inf (inf–inf)
VIL	inf (11.0–inf)	inf (15.7–inf)	inf (15.7–inf)	26.5 (4.4–67.9)
TAN	6.7 (2.3–20.9)	7.1 (2.8–15.8)	6.7 (3.0–12.1)	5.1 (2.1–9.5)

**Table 5:** Descriptive statistics of mitochondrial sequences’ (concatenated: COI + Cytb) variability. Sample code: n = number of sequences; nh = number of haplotypes; hd = haplotype diversity and relative standard deviation; π = nucleotide diversity and relative standard deviation. Codes are in accordance with those in Table 1.

Code	n	nh	hd	π
CAG	36	32	0.992 ± 0.009	0.007 ± 0.001
MAL	40	37	0.995 ± 0.007	0.009 ± 0.001
VIL	38	38	1.000 ± 0.006	0.010 ± 0.001
TAN	29	9	0.709 ± 0.081	0.003 ± 0.001
Wild	114	107	0.942 ± 0.006	0.009 ± 0.000
Overall	143	107	0.984 ± 0.006	0.009 ± 0.000

**Table 6:** Results of the neutrality tests and mismatch analyses (concatenated sequences): D = Tajima D test; FS = Fu’s FS test; R2 = Ramos-Onsins and Rozas’s R2 index; Harpending’s raggedness index (Hri) and the sum of squared deviations (SSD) were estimated under the sudden demographic expansion model (dem) and the spatial expansion model (spa). In bold are the statistical values significant at a level of 5%. Fu’s FS was considered significant only when the *p*-value was below 0.02. Wild = CAG + MAL + VIL.

Sample	D	FS	R2	SSDdem	Hri_dem	SSDspa	Hri_spa
CAG	−1.257	**−18.516**	**0.070**	0.015	0.090	0.157	0.420
MAL	−0.964	**−20.735**	0.081	0.007	0.410	0.009	0.420
VIL	−1.135	**−26.917**	0.074	0.107	0.940	0.003	0.950
Wild	**−1.470**	**−33.073**	**0.052**	0.007	0.730	0.116	0.150

## Data Availability

All the COI and Cytb haplotypes have been deposited in GenBank. Further details are available in the Supplementary Materials available online.

## Supplementary Materials

The following supporting information can be downloaded at: https://www.mdpi.com/article/10.3390/ani15040554/s1, Supplementary information on laboratory procedures. Supplementary Tables: Table S1, List of primers used; Table S2, Summary statistics for the eight microsatellite loci; Table S3, Summary statistics for the eight microsatellite loci; Table S4, Pairwise fixation indices calculated using FreeNA; Table S5, Results of the analysis of molecular variance (AMOVA) for the Sardinian sites; Table S6, Results of the Kruskal–Wallis nonparametric statistical tests; Table S7, List of COI sequences; Table S8, List of Cytb sequences; Table S9, List of the combined COI + Cytb sequences; Table S10, Descriptive statistics of mitochondrial sequences variability; Table S11, Results of the analysis of molecular variance (AMOVA) for the COI and Cytb sequences; Table S12, Pairwise fixation indices Φ_ST_. Supplementary Figures: Figure S1, Migration rates; Figure S2, Mismatch distributions; Figure S3, Haplotype network of the COI sequences; Figure S4, Geographical distribution of the BAPS haplogroups (COI sequences); Figure S5, COI sequences: pairwise *F*_ST_; Figure S6, Haplotype network of the Cytb sequences; Figure S7, Geographical distribution of the BAPS haplogroups (Cytb sequences). Figure S8, Cytb sequences: pairwise *F*_ST_. References [111,112,113,114,115,116,117,118,119,120,121] are cited in the supplementary materials.
