# Supplementary material for: The More the Better: Genetic Monitoring of Paracentrotus lividus (Lamarck, 1816) Experimental Restockings in Sardinia (Western Mediterranean Sea)
Source: Animals (Basel). 2025 Feb 14;15(4):554. doi: 10.3390/ani15040554 (PMC11852012; doi:10.3390/ani15040554)
Supplement: Supplementary file 1 [file animals-15-00554-s001.zip › Plividus_supplementary.pdf]

Article

The more the better: genetic monitoring of *Paracentrotus lividus* (Lamarck, 1816) experimental restockings in Sardinia (western Mediterranean Sea).

Simone Di Crescenzo[1†], Chiara Pani[1†], Viviana Pasquini[1], Marco Maxia[2], Pierantonio Addis[1*] and Rita Cannas [1,*]

Supplementary Material

1 Supplementary Information

1.1 Larval rearing

Fifty wild sea urchins broodstock were collected from the western coast of the Gulf of Cagliari (south of Sardinia 39°06'48"N 9°00'48"E) in November 2021 and were kept in flow-through seawater tanks for 24h before the spawning induction. *P. lividus* were induced to spawn by injection of 1M KCl (40 μl per g of body weight) into the coelom via the peristomial membrane. Females (n=10) released the eggs, and each batch was fertilized by adding few drops of diluted sperm from three random males (n=20) for each batch. The occurrence of fertilization was assessed after 2 h, and after 24h embryos were moved to six hatchery tanks (150 L water volume each) containing about 600.000 embryos each. Larval rearing was carried out according to [1] and [2]. After 28 days, larvae reached the competence for the settlement and were then moved in the 700-L settlement tank previously conditioned with microalgae biofilm to foster the larval metamorphosis. Once post-larvae became visible juveniles they were fed with the green seaweed *Ulva sp.*, collected in the wild. Juveniles were left in a growing tank until reaching an average test diameter of 15-20 mm and then collected for the genetic analysis.

1.2 Laboratory procedures

Tissue samples from the perioral membrane of the Aristotle's lantern were obtained from purple sea urchins collected during scientific surveys or at a farming facility and preserved in 2.5 mL cryovial tubes containing about 1.5 mL of 95% ethanol at -18°C until DNA extraction was performed.

1.3 Microsatellites amplification

Paracentrotus lividus individuals were genotyped at nine microsatellite loci, described in [3]: Pliv_Hist, Pliv_T, Pliv_15, Pliv_28, Pliv_F, Pliv_C, P_Liv_B, Pliv_32, Pliv_L.

Primer pairs are described in Table S1.

The nine mitochondrial markers were divided into two mixes: the mix A containing the loci Pliv_32, Pliv_F, Pliv_L, Pliv_B, and Pliv_C, and mix B containing the loci Pliv_Hist, Pliv_T, Pliv_28 and Pliv_15. PCR reactions were performed in a final volume of 15 μL containing 1X Wonder Taq Hot Start Reaction buffer, 0.5 pmol of each primer, 5 ng/L of gDNA, and 0.75 U of

WonderTaq DNA polymerase. PCR conditions consisted of an initial denaturation of 5 min of denaturation at 95 °C, followed by 45 cycles of 0.45 s at 95 °C, 60 s at 58 °C and 0.45 s at 72 °C, and a final extension step for 10 min at 72 °C. The quality of extracted DNA and PCR outcomes were evaluated on 2,5% agarose gel electrophoresis.

Amplicons were sized at Macrogen Korea using the GS-600LIZ internal size standard.

1.4 Mitochondrial gene amplification

The primers for the amplifications of Cytb and COI were obtained from Maltagliati [4], and [5], respectively (see Table S1 for details).

PCR reactions for Cyt-b mtDNA were performed in 25 μL total volume containing 1× DreamTaq buffer, 2 mM of MgCl, 0.2 mM of dNTPs, 0.8 pmol of each primer and 1U of DreamTaq DNA polymerase (Thermo Fisher Scientific, Waltham, MA, USA). Similarly, COI fragment was amplified in a 25 μL total volume PCR using 1x WonderTaq buffer, 0.4 pmol of each primer, and 1 U of WonderTaq DNA polymerase. The amplifications were performed in a Mastercycler EP Gradient S Eppendorf.

CytB marker amplifications were performed with an initial denaturation of 2 min at 94 °C, 5 cycles of 30 s at 95 °C, 30 s at 53 °C (reduced by 1°C at each cycle), and 2 min at 72 °C, followed by 30 cycles of 30 s at 95 °C, 30 s at 48 °C, and a final extension step of 5 min at 72 °C. Similarly, a fragment of the COI gene was amplified for all specimens. PCR conditions consisted of an initial denaturation of 2 min at 95 °C, followed by 35 cycles of 30 s at 95 °C, 30 s at 51 °C and 1 min at 72 °C, and a final extension step for 10 min at 72 °C. The quality of extracted gDNA and PCR outcomes were evaluated on 1,5% agarose gel electrophoresis. All the amplicons were Sanger-sequenced using the same forward primers used during the amplification by the external provider Macrogen Europe (Milano, Italy).

1.5 Genetic differentiation and population structure

The software Arlequin v3.5 [6] was used to estimate pairwise Fixation Indices (Φ_{ST}) values were calculated from sequence divergences using the best models identified with MEGA (COI: T92+G+I [7]; Cytb and concatenated sequences: TN93+G [8]).

Bayesian multi-locus clustering was performed with v.6 (Bayesian Analysis of Population Structure) [9-11].

The clustering analysis was performed, considering the upper limit of K = 10, and 5 repetitions for each K. Using the groups identified in the mixture analysis, the admixture analysis was realised with 1000 realisations from the posterior of the allele frequencies.

For mitochondrial data, BAPS was run using the "clustering for linked loci" method, a codon model with five independent runs and setting the maximum number of clusters (K) to 10.

Finally, as identification of genetic structure in BAPS relies on Hardy-Weinberg Equilibrium optimization, and some of the sampling sites in our study were not in HWE, we obtained an additional representation of the genetic structure using the discriminant analysis of principal components (DAPC) [12]. This multivariate descriptive method is not dependent on any model assumption and thus can provide a useful validation of Bayesian clustering output describing clusters of genetically related individuals, maximizing the

genetic variation between groups. We used the R package Adegenet v. 2.1.10 [13,14] implemented in R v. 4.3.1(R Core Team, 2021) to carry out standard DAPC. The results of the analysis were presented graphically along the first and the second axes, according to the highest Eigenvalues. The number of PCs retained in the DAPC was chosen using cross-validation; it corresponds to the number of PCs associated with the lowest Mean Squared Error.

1.6 Demography and connectivity

The program BOTTLENECK [15] measures heterozygosity excess with respect to the mutation-drift equilibrium characteristic of population-size reductions for each population to infer recent population bottlenecks. The two-phase model (TPM) was used in BOTTLENECK with the following parameters: multi-step changes = 90%, variance = 12. Significance for heterozygosity excess was checked using the one-tailed Wilcoxon's signed rank-test.

Settings in NEESTIMATOR [16] considered the screening out of rare alleles by using the critical allele frequency value of Pcrit = 0.05, 0.02 and 0.01 [17]. The upper and lower bounds of 95% confidence intervals for Ne were calculated using the jackknife option in order to reduce the potential bias associated with the estimation of confidence intervals approach to the linkage disequilibrium method.

The outputs of *DivMigrate* [18] are plotted in a network where populations are nodes connected by links (if gene flow is detected), with closely related nodes being plotted closer in the network space. Computations were performed, implementing using G_{ST} as index of differentiation with an arbitrary filter threshold set at 0.3 to retain informative connectivity values. G_{ST} was chosen since it is similar to the F_{ST} values applied above [19], and also because G_{ST} values could be interpreted as a measure of the level of allele fixation in populations and also reflect the population's demographic properties, e.g., the number of migrants per generation under assumed mutation rate [20]. Asymmetric gene flow between all pairs of populations being tested using 10, 000 bootstrap replicates.

The Coalescent Bayesian Skyline plot (BPS) was realized with Beast v.1.10.4 [21]. The analysis was conducted running 50,000,000 MCMC generations sampled every 5000 generations with a 20% burn-in and mutation rate according to ([22] and references therein) and using TN93+G+I for the concatenated dataset (according to Smart Model Selection outputs; [23]). Log and tree files were analyzed with Tracer v.1.7.2 [24].

Supplementary Figures and Tables

Supplementary Tables

Table S1 List of primers used with specific details on sequence, mix, label and reference

Oligo Name	5` - Oligo Seq - 3`	mix	Label 5’	reference
**Pliv_B_F**	CCATCCTCTCTTGCGACTTC	A	VIC	[3]
**Pliv_B_R**	ACGGGGTCTTGATGTCAGTT	A		[3]
**Pliv_C_F**	GCGGGTGTGTCCTGTAAAGT	A	6-FAM	[3]
**Pliv_C_R**	GACAAGCAAAAAGTGGCACA	A		[3]
**Pliv_F_F**	CCATCCCTCTTGACTATCGTT	A	6-FAM	[3]
**Pliv_F_R**	AATGAATAGAGTGGGGACAGG	A		[3]
**Pliv_L_F**	TATTGCGCATGAGTCAGCTT	A	PET	[3]
**Pliv_L_R**	CGACTATCACAGCTCGCATT	A		[3]
**Pliv_T_F**	AAAGCGAGAACGGATGACTG	B	VIC	[3]
**Pliv_T_R**	CTCTCCGTGTACGTCTGTCG	B		[3]
**Pliv_Hist_F**	ATGCACAAACGGCTCTTTTC	B	6-FAM	[3]
**Pliv_Hist_R**	TTGCACGTTGTTCATTGTCA	B		[3]
**Pliv_15_F**	ACCGCCCTTTAATCTGTCTC	B	NED	[3]
**Pliv_15_R**	GAGTGGCTACGAGAGAGTGG	B		[3]
**Pliv_28_F**	TGTATGTTCGCTCGGACTTG	B	PET	[3]
**Pliv_28_R**	GTATTGCCACACGACTCTCG	B		[3]
**Pliv_32_F**	TCAAAATACCACACACTATTTTTGC	A	NED	[3]
**Pliv_32_R**	AGCTGCAGTCACACTCATGC	A		[3]
**cytB_Pliv_F**	TTAACACAAGAATTAAATACAACGCC			[4]
**cytB_Pliv_R**	CTCTCTTTGAAGCATTTTCAGTGC			[4]
**COI_Pliv_F**	ATAATGATAGGAGGRTTTGG			[5]
**COI_Pliv_R**	GCTCGTGTRTCTACRTCCAT			[5]

Table S2 Summary statistics for the eight microsatellite loci used in studying *P. lividus* samples. n = total number of alleles per locus, Na = mean number of alleles per sample; Ar = allelic richness; PAr= private allelic richness; Fnu = null allele frequence; Ho = observed heterozygosity; uHe= corrected expected heterozygosity; F_{IS} = inbreeding fixation index; HWE = probability values for Hardy-Weinberg equilibrium test. Values statistically significant after FDR correction are indicated in bold.

	**Pliv_Hist**	**Pliv_T**	**Pliv_15**	**Pliv_28**	**Pliv_C**	**P_Liv_B**	**Pliv_32**	**Pliv_L**
**n**	37	21	29	26	31	22	20	29
**Na**	22.500	13.500	20.000	17.750	18.000	15.000	13.750	17.500
**Ar**	20.945	13.193	19.221	17.153	16.712	14.278	13.181	17.280
**PAr**	2.003	1.978	1.399	0.755	2.291	1.117	1.042	1.604
**Fnu**	0.206	0.187	0.222	0.239	0.177	0.216	0.285	0.471
**Ho**	0.491	0.601	0.504	0.531	0.536	0.517	0.359	0.102
**uHe**	0.866	0.892	0.929	0.916	0.876	0.867	0.871	0.880
**F_{IS}**	**0.438**	**0.302**	**0.461**	**0.406**	**0.396**	**0.379**	**0.590**	**0.872**
**HWE**	***	***	***	***	***	***	***	***

Table S3 Summary statistics for the eight microsatellite loci in each sample used in studying *P. lividus* samples. Acronyms are consistent with Table 1. n = number of individual genotyped, Na = number of alleles; Ar = allelic richness; PAr= private allelic richness; Fnu = null allele frequence; Ho = observed heterozygosity; uHe= corrected expected heterozygosity; F_{IS} = inbreeding fixation index; HWE = probability values for Hardy-Weinberg equilibrium test. Values statistically significant are indicated in bold. *** = p<0.001, ns = not significant.

Sample	Locus	n	Na	Ar	Par	Fnu	Ho	uHe	F_{IS}	HWE
**CAG**	**Pliv_Hist**	45	29	26.533	2.154	0.132	0.689	0.959	**0.284**	ns
	**Pliv_T**	45	15	14.437	1.730	0.147	0.622	0.908	**0.317**	***
	**Pliv_15**	45	22	20.897	2.354	0.243	0.467	0.949	**0.511**	***
	**Pliv_28**	44	23	21.630	2.387	0.186	0.614	0.941	**0.351**	***
	**Pliv_C**	45	24	22.166	5.594	0.118	0.711	0.935	**0.241**	***
	**P_Liv_B**	42	16	15.303	0.149	0.329	0.381	0.912	**0.585**	***
	**Pliv_32**	44	16	15.262	2.084	0.300	0.364	0.906	**0.602**	***
	**Pliv_L**	36	23	22.885	2.188	0.525	0.167	0.953	**0.827**	***
**MAL**	**Pliv_Hist**	38	27	26.178	2.674	0.209	0.579	0.949	**0.393**	***
	**Pliv_T**	35	15	15.000	3.222	0.262	0.600	0.918	**0.350**	**
	**Pliv_15**	39	25	24.055	3.126	0.193	0.564	0.954	**0.412**	***
	**Pliv_28**	35	21	21.000	0.618	0.255	0.629	0.945	**0.338**	***
	**Pliv_C**	39	21	19.961	3.094	0.142	0.590	0.881	**0.334**	***
	**P_Liv_B**	38	18	17.500	0.834	0.247	0.474	0.907	**0.481**	***
	**Pliv_32**	39	15	14.849	0.340	0.200	0.513	0.900	**0.433**	***
	**Pliv_L**	37	21	20.820	3.223	0.459	0.108	0.947	**0.887**	***
**TAN**	**Pliv_Hist**	48	6	5.711	0.417	0.252	0.208	0.612	**0.662**	***
	**Pliv_T**	48	9	8.657	0.011	0.127	0.583	0.818	**0.289**	***
	**Pliv_15**	48	11	10.581	0.013	0.296	0.313	0.867	**0.642**	***
	**Pliv_28**	47	9	8.474	0.001	0.268	0.383	0.833	**0.543**	***
	**Pliv_C**	48	8	7.653	0.020	0.244	0.333	0.777	**0.574**	***
	**P_Liv_B**	47	7	6.747	0.000	0.089	0.681	0.726	0.063	***
	**Pliv_32**	47	10	9.425	0.008	0.366	0.170	0.799	**0.789**	***
	**Pliv_L**	47	7	6.663	0.002	0.370	0.085	0.674	**0.875**	***
**VIL**	**Pliv_Hist**	47	28	25.358	2.767	0.231	0.489	0.945	**0.485**	***
	**Pliv_T**	45	15	14.677	2.951	0.211	0.600	0.926	**0.355**	***
	**Pliv_15**	46	22	21.352	0.103	0.156	0.674	0.948	**0.291**	***
	**Pliv_28**	46	18	17.507	0.016	0.246	0.500	0.946	**0.474**	***
	**Pliv_C**	47	19	17.068	0.456	0.204	0.511	0.909	**0.441**	***
	**P_Liv_B**	47	19	17.563	3.487	0.199	0.532	0.922	**0.426**	***
	**Pliv_32**	46	14	13.190	1.738	0.275	0.391	0.879	**0.558**	***
	**Pliv_L**	41	19	18.753	1.003	0.531	0.049	0.945	**0.949**	***

Table S4. Pairwise fixation indices calculated for microsatellite loci using FreeNA: FST not corrected (below the diagonal) and corrected with ENA method for null alleles (above the diagonal). In bold the significant values.

F_{ST}\ F_{ST} ENA	CAG	MAL	TAN	VIL
CAG		0	**0.069**	0.002
MAL	0		**0.067**	0.003
TAN	**0.091**	**0.086**		**0.073**
VIL	0.001	0.003	**0.094**	

Table S5. Results of the analysis of molecular variance (AMOVA) for the Sardinian sites. Samples were grouped according to the results of BAPs and/or DAPC.

**AMOVA**	**% Variance**	**Fixation Index**	**p-value**
**1 group: (TAN+CAG+MAL+VIL)**			
**microsatellites**			
Among groups	5.4	F_{ST} = 0.054	0.000
Within populations	94.6		
**1 group: (TAN+CAG+MAL+VIL)**			
**COI+Cytb**			
Among groups	21.8	Φ_{ST}= 0.218	0.00
Within populations	78.2		
**2 groups: (TAN/CAG+MAL+VIL)**			
**microsatellites**			
Among groups	8.81	F_{CT} = 0.088	ns
Among populations	0.51	F_{SC} = 0.006	ns
Within populations	90.64	F_{ST} = 0.094	0.00
**2 groups: (TAN/CAG+MAL+VIL)**			
**COI+Cytb**			
Among groups	17.99	Φ_{CT}= 0.180	ns
Among populations	12.49	Φ_{SC}= 0.152	0.00
Within populations	69.52	Φ_{ST}= 0.305	0.00
**3 groups: (TAN/CAG+MAL/VIL)**			
**microsatellites**			
Among groups	6.05	F_{CT} = 0.061	ns
Among populations	0.19	F_{SC} = 0.002	ns
Within populations	93.76	F_{ST} = 0.062	0.000
**3 groups: (TAN/CAG/MAL+VIL)**			
**COI+Cytb**			
Among groups	27.46	Φ_{CT}= 0.275	ns
Among populations	-0.64	Φ_{SC}= -0.009	ns
Within populations	73.19	Φ_{ST}= 0.268	0.00

Table S6 Results of the Kruskal-Wallis nonparametric statistical tests, nloci= n° loci included in the comparison, p-value for the comparison involving Ar= Allelic richness, Na = mean number of alleles, Ho = observed hererozigosity, He = expected heterozygosity, F_{IS} = inbreeding index.

	**nloci**	**Ar**	**Na**	**Ho**	**He**	**F_{IS}**
**[25]** vs present study (wild samples)	5	\	0.480	**0.016**	0.166	**0.018**
**[26]** vs present study (wild samples)	6	\	0.919	**0.003**	0.064	**0.003**
**[27]** vs present study (wild samples)	8	0.332	\	**0.036**	**0.036**	**0.035**
**[28]** vs present study (wild samples)	7	0.855	\	0.058	0.195	0.060

Table S7. List of COI sequences utilized in the current study. The table shows: Area, Sample Code, Individual Code, NCBI AccNum, Region, MacroRegion, Haplotype Code, Reference. In bold the haplotypes newly observed.

SEE Excel File

Table S8. List of Cytb sequences utilized in the current study. The table shows: Area, Sample Code, Individual Code, NCBI AccNum, Region, MacroRegion, Haplotype Code, Reference. In bold the haplotypes newly observed.

SEE Excel File

Table S9. List the combined COI+Cytb sequences utilized in the current study. The table shows: Area, origin (w = wild, f = farmed), Sample Code, individual Code, Concatenate_Hap_Code

SEE Excel File

Table S10. Descriptive statistics of mitochondrial sequences variability; sample code, n = number of sequences; nh= number of haplotypes; hd = haplotype diversity and relative standard deviation, π = nucleotide diversity and relative standard deviation. Codes are in accordance with Table 1.

code	n	nh	hd	π
**COI**				
**CAG**	48	22	0.938±0.018	0.006±0.000
**MAL**	45	25	0.908±0.001	0.006±0.001
**VIL**	48	33	0.975±0.000	0.008±0.001
**TAN**	48	5	0.589±0.066	0.002±0.001
**Wild**	141	59	0.951±0.009	0.007±0.000
**overall**	189	60	0.939±0.008	0.007±0.000
**Cytb**				
**CAG**	36	22	0.919±0.037	0.007±0.001
**MAL**	43	30	0.979±0.010	0.010±0.001
**VIL**	38	30	0.979±0.014	0.011±0.001
**TAN**	29	7	0.377±0.013	0.004±0.002
**Wild**	117	65	0.972±0.008	0.012±0.000
**overall**	146	69	0.925±0.018	0.010±0.000

Table S11. Results of the analysis of molecular variance (AMOVA) for the COI and Cytb sequences. Samples were grouped according to the main biogeographic regions (see Table S6 and S7 for Codes and details). COI- Atlantic: (ROS. TAR. SAN. TEN); Mediterranean: (MED. HER. FER. IBZ. CPA. CDG. CAS. BLA. MAL. CAG. VIL. EGY). Cytb- Atlantic: (GAL. ROS. BAI. MOS); WMediterranean: (MED. PAL. QUE. PIT. ALG. CDE. UST. CAG. MAL. VIL. TUN); Adriatic: (BRI. MAN. LES. ANC. MLJ. MIR. NAX. SCN); CWMediterranean: MLT. EPA. KYR. RHO)

AMOVA	% Variance	Fixation Index	p-value
**1 group**			
**COI**			
Among groups	21.47	Φ_{ST}= 0.215	0.00
Within populations	78.53		
**2 groups: Mediterranean vs Atlantic**			
**COI**			
Among groups	10.72	Φ_{CT}= 0.107	0.002
Among populations	5.57	Φ_{SC}= 0.062	0.000
Within populations	83.71	Φ_{ST}= 0.163	0.000
**1 group**			
**Cytb**			
Among groups	23.42	Φ_{ST}= 0.234	0.00
Within populations	76.58		
**4* groups: Atlantic/WMediterranean/EMediterranean/Adriatic**			
**Cytb**			
Among groups	16.29	Φ_{CT}= 0.163	0.000
Among populations	6.28	Φ_{SC}= 0.075	0.000
Within populations	77.42	Φ_{ST}= 0.226	0.000
**5* groups: Atlantic/WMediterranean/CAG/EMediterranean/Adriatic**			
**Cytb**			
Among groups	19.61	Φ_{CT}= 0.196	0.000
Among populations	2.47	Φ_{SC}= 0.031	0.016
Within populations	77.92	Φ_{ST}= 0.221	0.000

Table S12. Pairwise fixation indices Φ_{ST} calculated for mitochondrial genes: COI (below the diagonal), and Cytb (above the diagonal). Negative values have been replaced with zero. The bold values are significant after FDR correction.

COI\Cytb	**CAG**	**MAL**	**VIL**	**TAN**
CAG		**0.216**	**0.242**	0.024
MAL	**0.072**		0	**0.343**
VIL	**0.119**	0.006		**0.374**
TAN	**0.313**	**0.451**	**0.460**	

Supplementary Figures

Figure S1 Migration rates of *Paracentrotus lividus* populations calculated with DivMigrate and based on G_{ST}. Acronyms are shown in Table 1. Arrows' thickness is proportional to migration level**.** Abbreviations are in accordance with Figure 1.

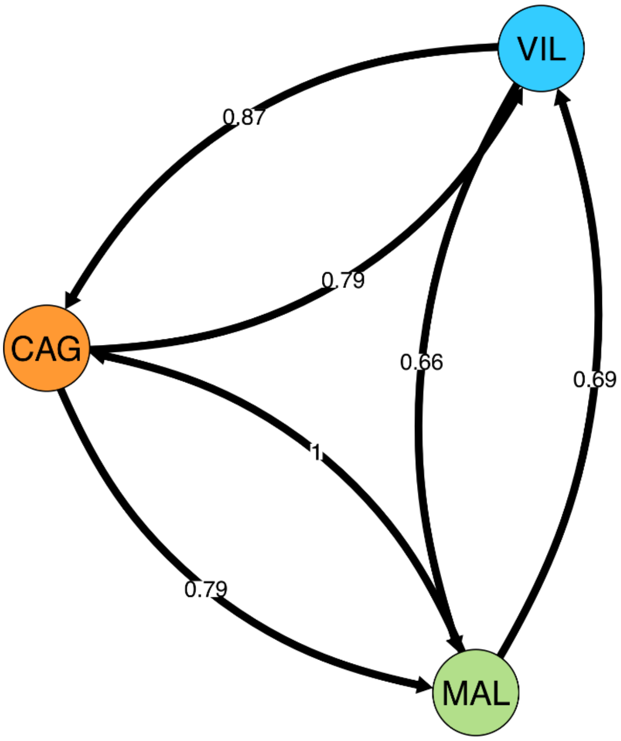

Figure S2 Histograms of mismatch distribution of COI+Cytb pairwise DNA sequence differences. Obs = observed frequencies. Exp c =expected under a constant model; Exp.gd = expected under a growth/decline model. Abbreviations are in accordance with Figure 1. wild=CAG+MAL+VIL

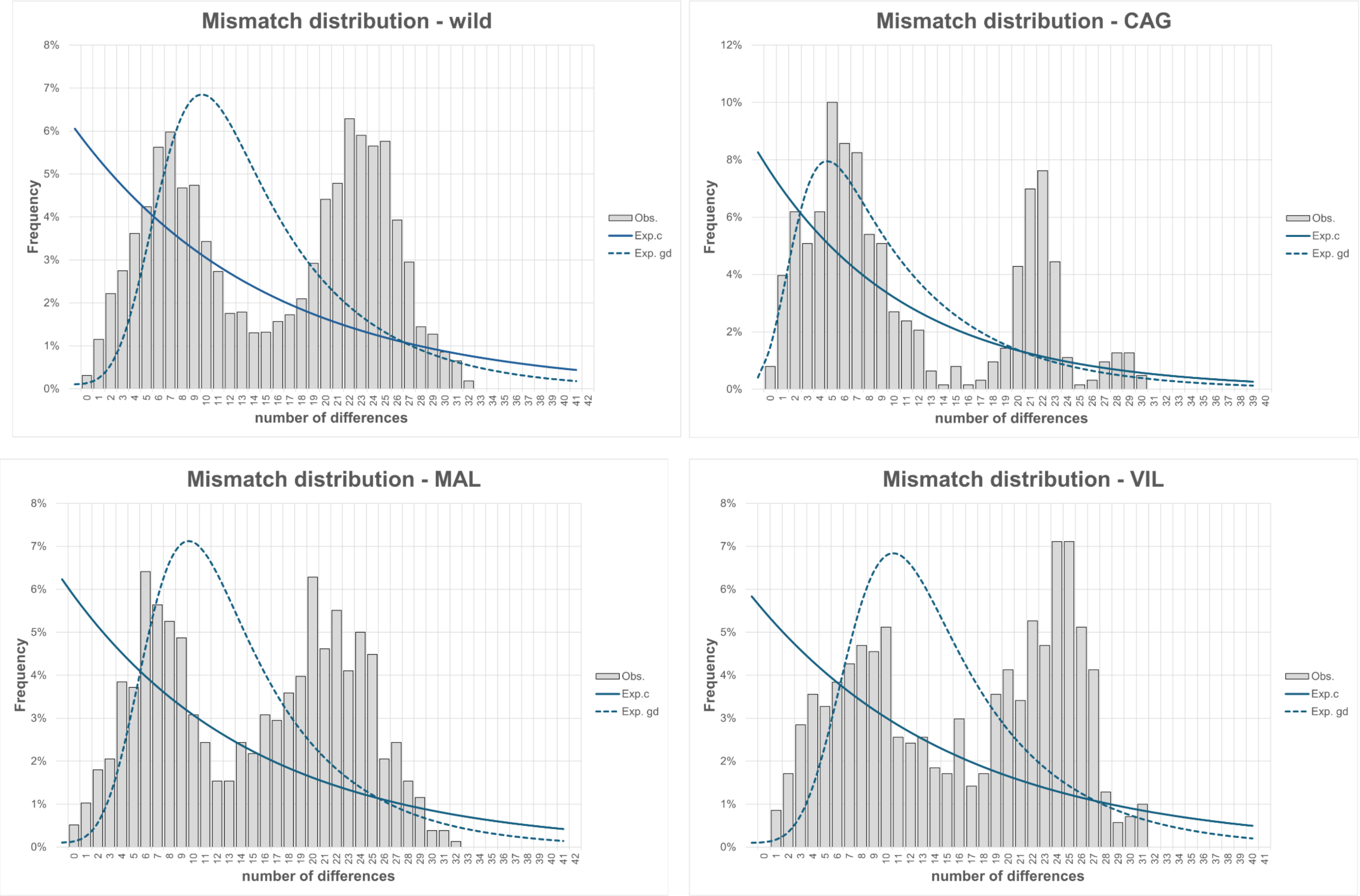

Figure S3 Haplotype network realized with the TCS method of the COI sequences. The size of pie charts is proportional to the corresponding haplotype frequency, while the colour indicates the geographical origin. Dots indicate inferred unsampled haplotypes, while lines single substitutions. Abbreviations are in accordance with Figure 1 and Table S7.

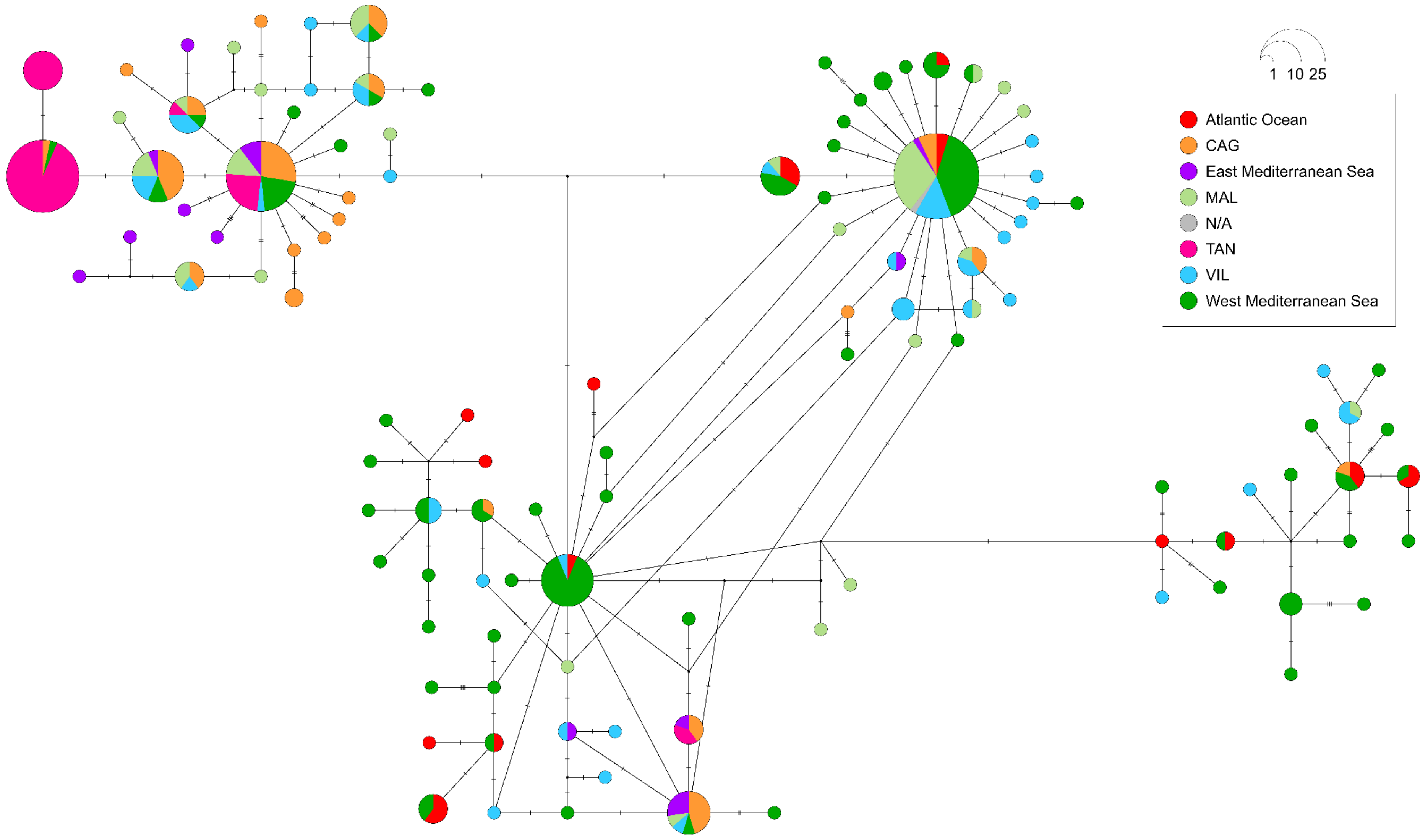

Figure S4 Geographical distribution of BAPS haplogroups (COI sequences). Chart size is drawn proportionally to the number of sequences in each location. The four haplogroups identified by BAPS are shown with different colours. Sample codes as in Table 1 and S7.

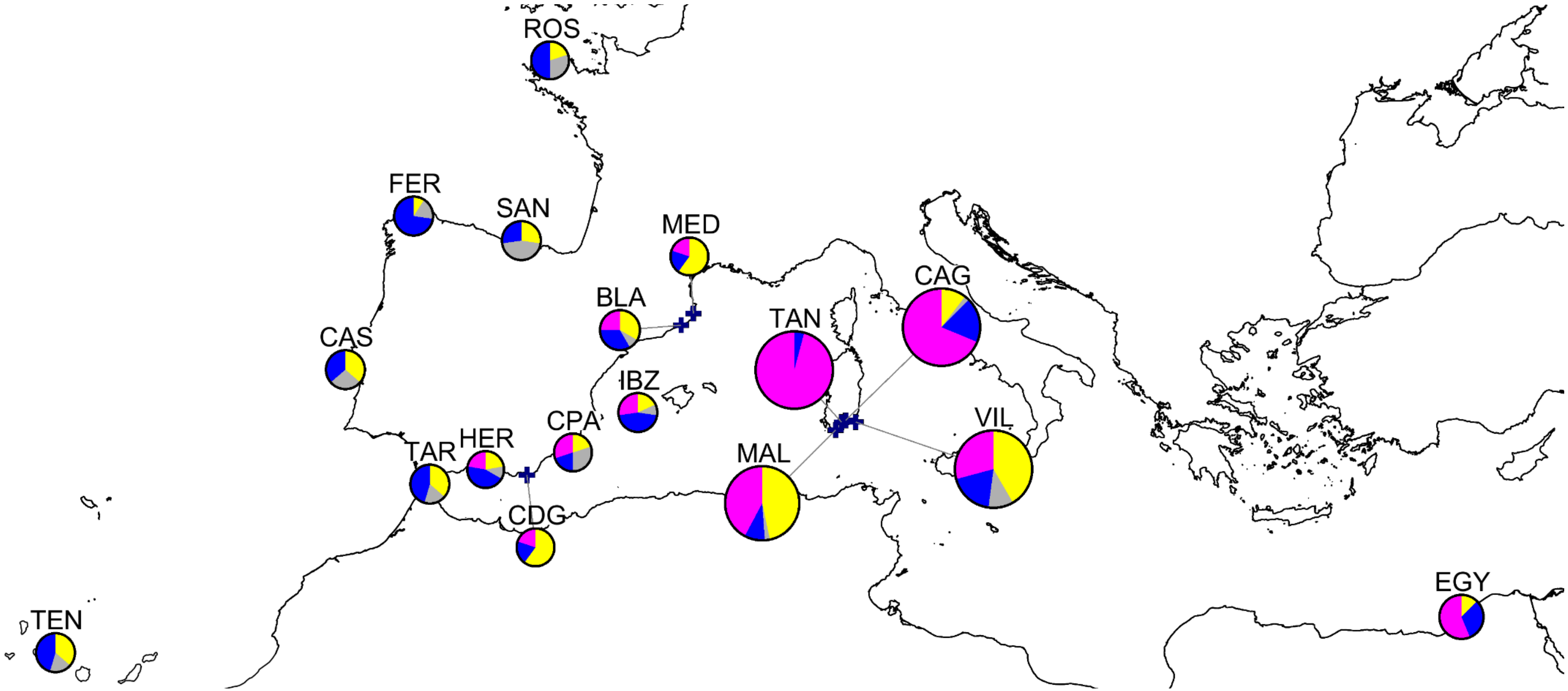

Figure S5 COI sequences: pairwise F_{ST} plot between sampling sites. Darker blue represents a higher pairwise F_{ST} value. and lighter blue represents a lower value. *** = p<0.001. ** = p<0.01. * = p< 0.05. Acronyms detailed in Table S7.

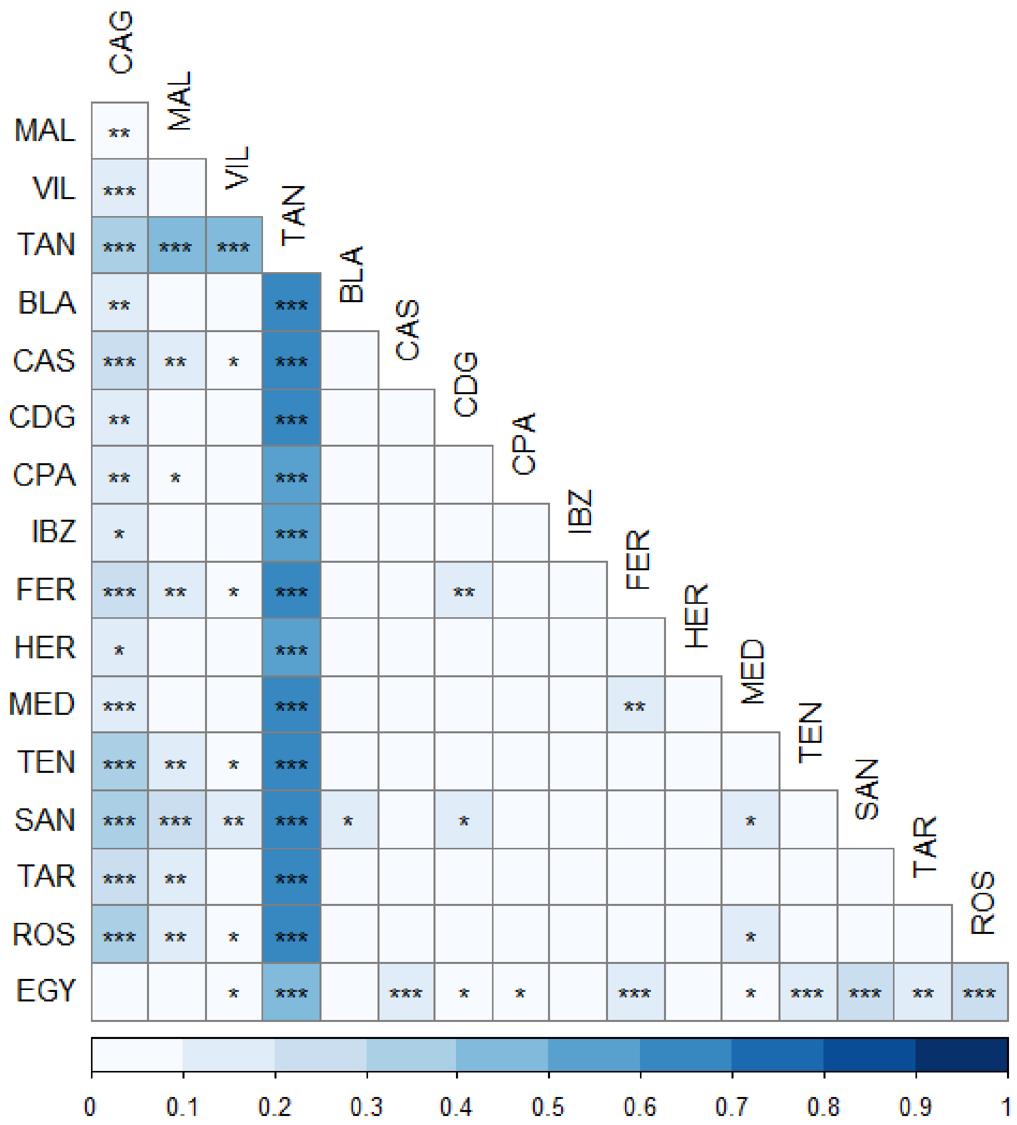

Figure S6 Haplotype network realized with the TCS method of the Cytb sequences. The size of pie charts is proportional to the corresponding haplotype frequency, while the colour indicates the geographical origin. Dots indicate inferred unsampled haplotypes, while lines single substitutions. Abbreviations are in accordance with Figure 1 and Table S8.

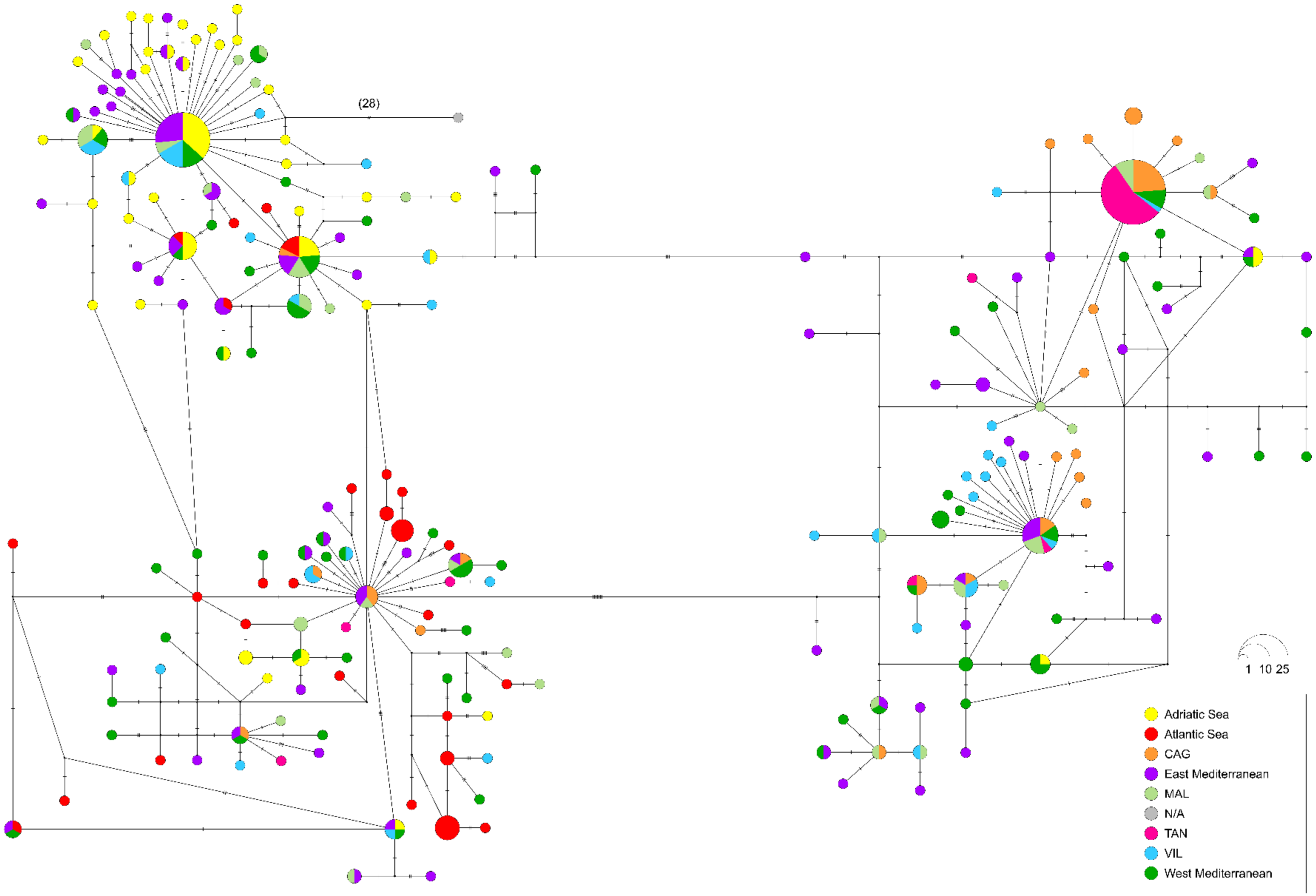

Figure S7 Geographical distribution of BAPS haplogroups (Cytb sequences). Chart size is drawn proportionally to the number of sequences in each location. The three haplogroups identified by BAPS are shown with different colours. Sample codes as in Table 1 and S8.

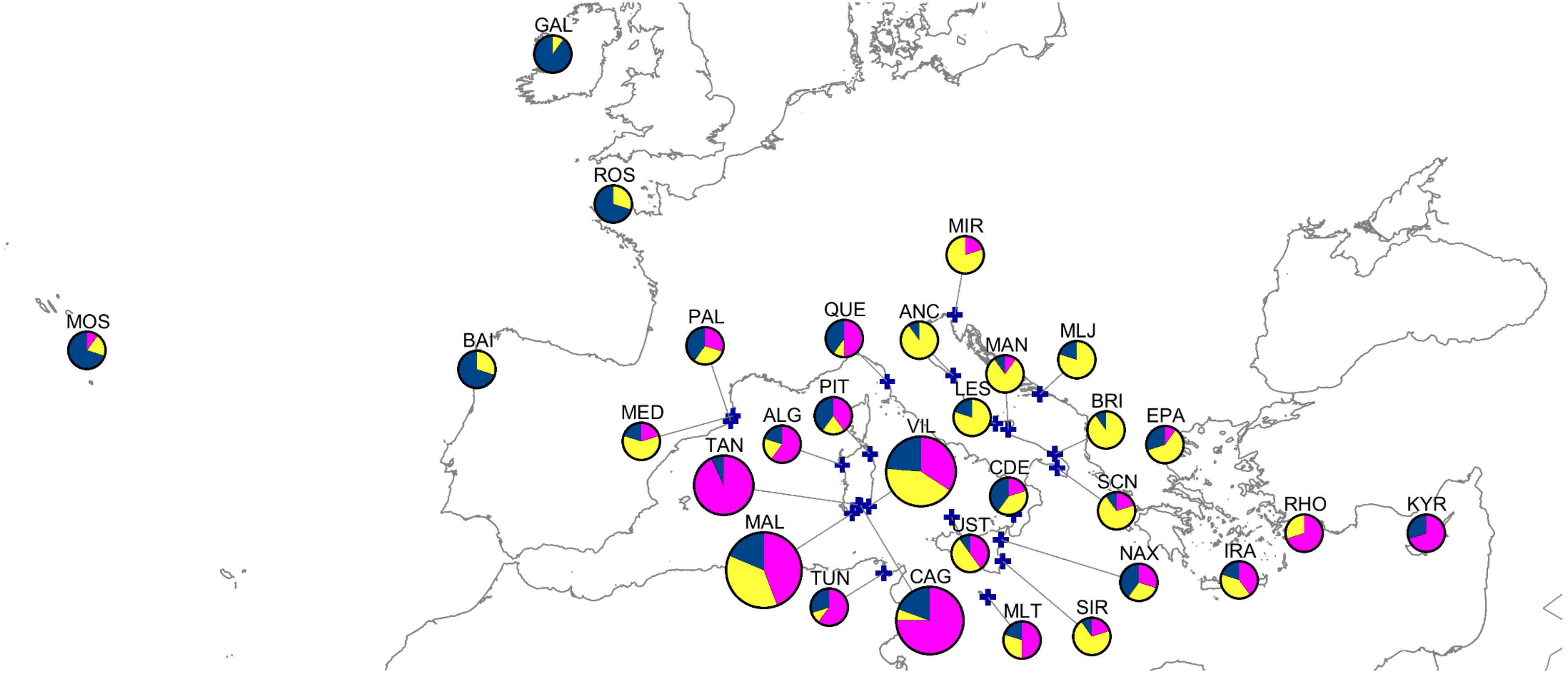

Figure S8 Cytb sequences: pairwise F_{ST} plot between sampling sites. Darker blue represents a higher pairwise F_{ST} value. and lighter blue represents a lower value. *** = p<0.001. ** = p<0.01. * = p< 0.05. Acronyms are fully explained in Table S8.

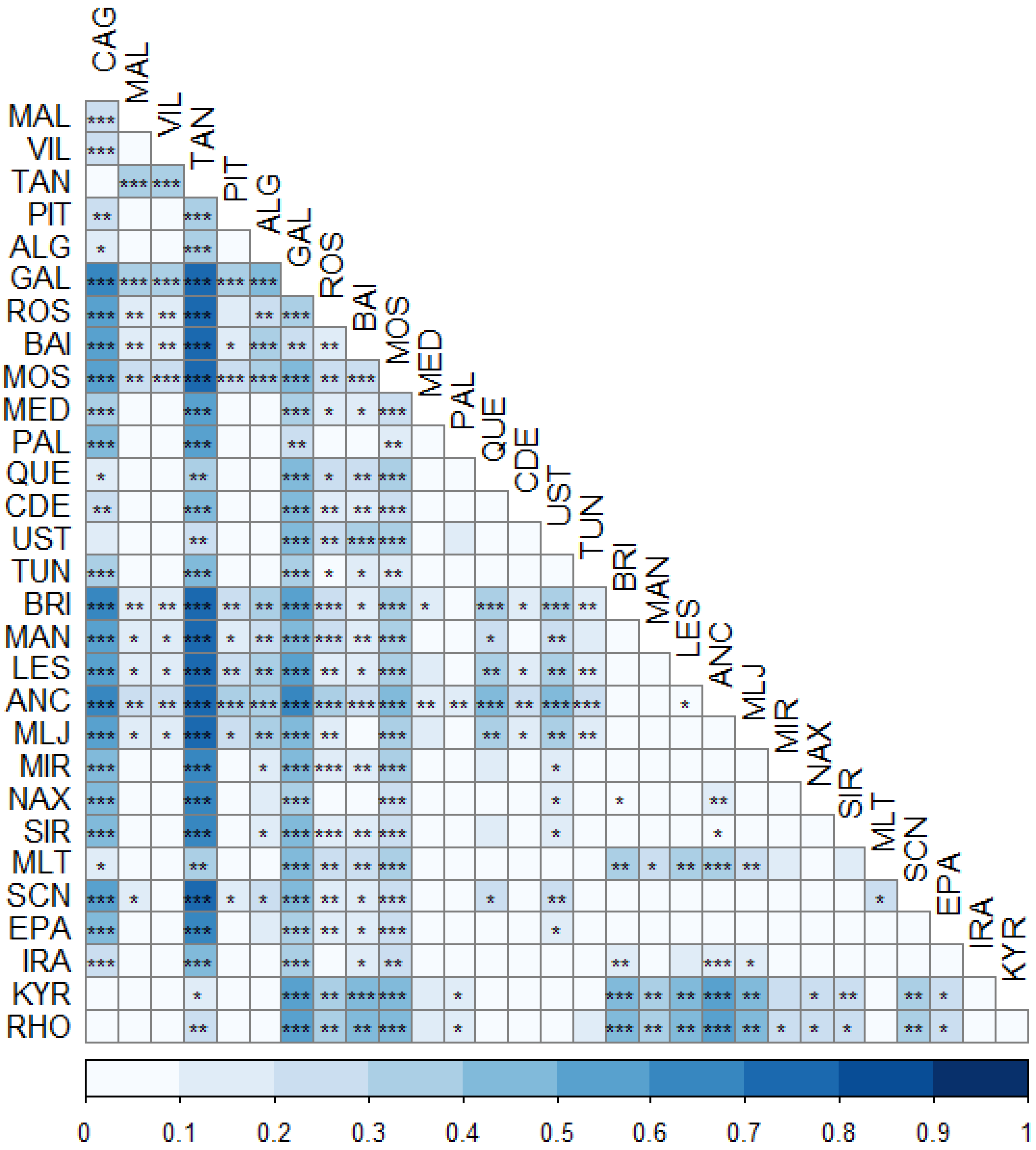

References

1. Carboni, S.; Kelly, M.S.; Hughes, A.D.; Vignier, J.; Atack, T.; Migaud, H. Evaluation of flow through culture technique for commercial production of sea urchin (*Paracentrotus lividus*) larvae. *Aquaculture Research* **2014**, *45*, 768-772, doi:10.1111/are.12019.
2. Giglioli, A.A.; Addis, P.; Pasquini, V.; Secci, M.; Hannon, C. First assessment of restocking efficacy of the depleted sea urchin Paracentrotus lividus populations in two contrasted sites. *Aquaculture Research* **2021**, *52*, 2896-2900, doi:10.1111/are.15098.
3. Calderón, I.; Turon, X.; Pascual, M. Isolation of nine nuclear microsatellites in the common Mediterranean sea urchin, *Paracentrotus lividus* (Lamarck). *Molecular Ecology Resources* **2009**, *9*, 1145-1147, doi:10.1111/j.1755-0998.2009.02585.x.
4. Maltagliati, F.; Di Giuseppe, G.; Barbieri, M.; Castelli, A.; Dini, F. Phylogeography and genetic structure of the edible sea urchin Paracentrotus lividus (Echinodermata: Echinoidea) inferred from the mitochondrial cytochrome b gene. *BIOLOGICAL JOURNAL OF THE LINNEAN SOCIETY* **2010**, *100*, 910-923, doi:10.1111/j.1095-8312.2010.01482.x.
5. Arndt, A.; Marquez, C.; Lambert, P.; Smith, M.J. Molecular Phylogeny of Eastern Pacific Sea Cucumbers (Echinodermata: Holothuroidea) Based on Mitochondrial DNA Sequence. *Molecular Phylogenetics and Evolution* **1996**, *6*, 425-437, doi:https://doi.org/10.1006/mpev.1996.0091.
6. Excoffier, L.; Lischer, H.E.L. Arlequin suite ver 3.5: a new series of programs to perform population genetics analyses under Linux and Windows. *Molecular Ecology Resources* **2010**, *10*, 564-567, doi:10.1111/j.1755-0998.2010.02847.x.
7. Tamura, K. Estimation of the number of nucleotide substitutions when there are strong transition-transversion and G+C-content biases. *Mol Biol Evol* **1992**, *9*, 678-687, doi:10.1093/oxfordjournals.molbev.a040752.
8. Tamura, K.; Nei, M. Estimation of the number of nucleotide substitutions in the control region of mitochondrial DNA in humans and chimpanzees. *Mol Biol Evol* **1993**, *10*, 512-526, doi:10.1093/oxfordjournals.molbev.a040023.
9. Corander, J.; Marttinen, P. Bayesian identification of admixture events using multilocus molecular markers. *Mol Ecol* **2006**, *15*, 2833-2843, doi:10.1111/j.1365-294X.2006.02994.x.
10. Corander, J.; Marttinen, P.; Mantyniemi, S. A Bayesian method for identification of stock mixtures from molecular marker data. *Fishery Bulletin* **2006**, *104*, 550-558.
11. Corander, J.; Marttinen, P.; Sirén, J.; Tang, J. Enhanced Bayesian modelling in BAPS software for learning genetic structures of populations. *BMC Bioinformatics* **2008**, *9*, 539, doi:10.1186/1471-2105-9-539.
12. Jombart, T.; Devillard, S.; Balloux, F. Discriminant analysis of principal components: a new method for the analysis of genetically structured populations. *BMC Genetics* **2010**, *11*, 94, doi:10.1186/1471-2156-11-94.
13. Jombart, T.; Ahmed, I. adegenet 1.3-1: new tools for the analysis of genome-wide SNP data. *Bioinformatics* **2011**, *27*, 3070-3071, doi:10.1093/bioinformatics/btr521.
14. Jombart, T. adegenet: a R package for the multivariate analysis of genetic markers. *Bioinformatics* **2008**, *24*, 1403-1405, doi:10.1093/bioinformatics/btn129.
15. Piry, S.; Luikart, G.; Cornuet, J.M. Computer note. BOTTLENECK: a computer program for detecting recent reductions in the effective size using allele frequency data. *Journal of Heredity* **1999**, *90*, 502-503, doi:10.1093/jhered/90.4.502.

16. Do, C.; Waples, R.S.; Peel, D.; Macbeth, G.M.; Tillett, B.J.; Ovenden, J.R. NeEstimator v2: re-implementation of software for the estimation of contemporary effective population size (Ne) from genetic data. *Molecular Ecology Resources* **2014**, *14*, 209-214, doi:https://doi.org/10.1111/1755-0998.12157.

17. Waples, R.S.; Do, C. Linkage disequilibrium estimates of contemporary N e using highly variable genetic markers: a largely untapped resource for applied conservation and evolution. *Evol Appl* **2010**, *3*, 244-262, doi:10.1111/j.1752-4571.2009.00104.x.

18. Keenan, K.; McGinnity, P.; Cross, T.F.; Crozier, W.W.; Prodöhl, P.A. diveRsity: An R package for the estimation and exploration of population genetics parameters and their associated errors. *Methods in Ecology and Evolution* **2013**, *4*, 782-788, doi:https://doi.org/10.1111/2041-210X.12067.

19. Whitlock, M.C. G'ST and D do not replace FST. *Mol Ecol* **2011**, *20*, 1083-1091, doi:10.1111/j.1365-294X.2010.04996.x.

20. Alcala, N.; Goudet, J.; Vuilleumier, S. On the transition of genetic differentiation from isolation to panmixia: what we can learn from GST and D. *Theor Popul Biol* **2014**, *93*, 75-84, doi:10.1016/j.tpb.2014.02.003.

21. Suchard, M.A.; Lemey, P.; Baele, G.; Ayres, D.L.; Drummond, A.J.; Rambaut, A. Bayesian phylogenetic and phylodynamic data integration using BEAST 1.10. *Virus Evolution* **2018**, *4*, doi:10.1093/ve/vey016.

22. Calderón, I.; Giribet, G.; Turon, X. Two markers and one history: phylogeography of the edible common sea urchin *Paracentrotus lividus* in the Lusitanian region. *Marine Biology* **2008**, *154*, 137-151, doi:10.1007/s00227-008-0908-0.

23. Lefort, V.; Longueville, J.-E.; Gascuel, O. SMS: Smart Model Selection in PhyML. *Molecular Biology and Evolution* **2017**, *34*, 2422-2424, doi:10.1093/molbev/msx149.

24. Rambaut, A.; Drummond, A.J.; Xie, D.; Baele, G.; Suchard, M.A. Posterior Summarization in Bayesian Phylogenetics Using Tracer 1.7. *Syst Biol* **2018**, *67*, 901-904, doi:10.1093/sysbio/syy032.

25. Duchaud, S.; Durieux, E.D.H.; Coupe, S.; Pasqualini, V.; Ternengo, S. Spatio-temporal patterns based on demographic and genetic diversity of the purple sea urchin *Paracentrotus lividus* in the area around Corsica (Mediterranean Sea). *Mediterranean Marine Science* **2018**, *19*, 620-641, doi:10.12681/mms.14184.

26. Couvray, S.; Miard, T.; Bunet, R.; Martin, Y.; Grillasca, J.-P.; Bonnefont, J.-L.; Coupé, S. Experimental Release of Juvenile Sea Urchins (*Paracentrotus lividus*) in Exploited Sites along the French Mediterranean Coast. *Journal of Shellfish Research* **2015**, *34*, 555-563, 559.

27. Parrondo, M.; López, S.; Uz, S.; Rodríguez, C.; Carrasco, J.F.; García-Flórez, L.; Borrell, Y.J. Genetic monitoring of the declining European stony sea urchin *Paracentrotus lividus* from the central Bay of Biscay (Asturias, northwest Spain) and attempts to restore its wild populations. *Aquatic Conservation-Marine and Freshwater Ecosystems* **2022**, *32*, 309-328, doi:10.1002/aqc.3766.

28. Segovia-Viadero, M.; Serrão, E.A.; Canteras-Jordana, J.C.; Gonzalez-Wangüemert, M. Do hatchery-reared sea urchins pose a threat to genetic diversity in wild populations? *Heredity* **2016**, *116*, 378-383, doi:10.1038/hdy.2015.109.
